# Plant community and structural pattern analyses of Abraham Sacred Forest in Amhara Regional State, northwest Ethiopia

**DOI:** 10.1371/journal.pone.0317245

**Published:** 2025-01-14

**Authors:** Getinet Masresha Kassa, Ayenew Lisanu Teka, Getahun Tassew Melese

**Affiliations:** 1 Department of Biology, University of Gondar, Gondar, Ethiopia; 2 Department of Forestry, University of Gondar, Gondar, Ethiopia; Makerere University, UGANDA

## Abstract

Owing to its topographic variations, Ethiopia is a biodiversity-rich country. However, the long-term degradation of resources has resulted in isolated forest patches largely around sacred places. Thus, this work was aimed to evaluate the plant community formation and structural dynamics of the Abraham Sacred Forest patch. Data were collected from 60 plots located on transect lines. Five subplots (4 m^2^), four at each corner and center, were set to collect juveniles’ data. Individuals of each species and cover abundance were recorded, and adults’ stem girth was measured. Hierarchical cluster analysis was used to identify plant communities. A Kruskal-Wallis followed by Tukey’s honestly significant difference test was performed to check the statistical significance among the plant communities. Shannon-Wiener diversity index, equitability index, and non-parametric species richness estimators were used to quantify species diversity, evenness, and richness, respectively. Structural parameters and size class ratios were used to analyze the vegetation structure and regeneration status. Seventy wood species, distributed in 62 genera and 38 families, were recorded. Fabaceae was the most species-rich (10 species) family. Three plant communities were identified. A Kruskal-Wallis test indicated that the community types showed significant differences (P < 0.05) with respect to altitude and slope. The density and basal area of the forest were 4580.4 ha^-1^ and 35.18 m^2^ha^-1^ respectively. The inverted J-shaped pattern in DBH classes implies a good reproduction status. However, importance value index and regeneration status analyses revealed that certain species, like *Astropanax abyssinicum* (Hochst. ex. A. Rich) Seem, *Myrica salicifolia* Hochst. ex. A. Rich and *Dombeya torrida* (G.F.Gmel) Bamps, require conservation priority.

## Introduction

Forests covering 31% areas of the world [1] provide ecosystem services, which can be expressed in terms of provisioning, regulating, cultural and supporting services [2]. However, deforestation, climate change, habitat and biodiversity loss are global challenges that decrease forest coverage. In developing nations, limited alternative sources of income and employment opportunities make people heavily depend on their natural environment [3, 4] such as use of forest products [3, 5]. The production, trading and consumption of forest products are encouraged by market forces such as price and demand [5]. Another factor for reliance on natural resources is subsistence agriculture with small holdings that urges them to encroach on natural habitats [6]. This ultimately results conflict with wildlife that causes significant damage to agricultural crops and livestock [7]. The dependence on the natural environment is further reinforced by the inadequate access to contemporary resources such as energy, and healthcare [3, 8]. Due to the reasons mentioned above, conservation of biodiversity is a challenging task.

Ethiopia’s geographical location, topographic and soil variability created a range of ecological regions allowing for the existence of 10 diverse vegetation types [9], spanning from afro-alpine to arid and semi-arid vegetation types [10]. This makes Ethiopia one of the top floral rich (fifth) countries in tropical Africa [11], estimated to encompass around 6,027 higher plant species, 10% of which are endemic [12]. Despite this, the biological resources of Ethiopia are currently under critical threat mainly due to rapid population growth, the livelihood of which mainly depends on natural resource products. This substantially drives the rapid decline of natural vegetation [13]. According to [1] report, the forest coverage of Ethiopia experienced a decline from 17.2% in 1990 to 16.5% in 2000 to 15.9% in 2010 and reaching 15.24% in 2020 with the consequences of soil erosion and reduced capacity for carbon sequestration. The depletion of the natural vegetation has also led to the threat of many plant species [14]. According to [15], large number of threatened endemic plant species (120) is identified in Ethiopia; several of these species (35) were from the dry evergreen Afromontane forests. The country is also recognized as one of African countries with a high proportion of potentially endangered species [16]. Recent study on global land plant distribution [17] identified the Ethiopian highlands as hotspots for numerous rare plant species. The speedy depletion of forest resources in Ethiopia has, thus, led to a substantial decline in biodiversity to the extent of local extinction [18]. As a result of early human settlement and rudimentary farming, the rate of deforestation is exceptionally aggravated in the northern highlands of Ethiopia [18, 19] where the study area is located.

As highlighted above, like in other developing nations, Ethiopians are, thus, highly reliant on their natural environment. In order to lessen the impact of the anthropogenic pressure on the natural ecosystems, the government shall encourage sustainable agricultural practices, such as agro-forestry and organic farming [20, 21]; law enforcement steps to reduce deforestation [22]; awareness raising program that highlight the benefits of forests, and the need for conservation [23], and provide alternative livelihood options [24] such as eco-tourism, non-timber forest product harvesting [22, 24]. The combination of these measures, tailored to specific regional and local contexts, is often effective in reducing anthropogenic pressures on forests.

Since Ethiopia is a topographically variable country, in addition to anthropogenic activities, forests are shaped by terrain variables [25] such as altitude, slope, and aspect [26, 27]. Changes in altitudes have corresponding changes in climate [28], leading to distinct ecological zones (vegetation belts) [29]. These belts are characterized by specific plant communities adapted to the prevailing conditions at different altitudes [28]. Slope influences plant distribution by affecting water drainage, soil characteristics, and exposure to sunlight [30]. Steep slopes have faster water runoff, resulting thinner soil with poor accumulation of nutrients, favoring plants adapted in drier conditions and nutrient-poor soils [30, 31]. Aspect influences plant distribution by affecting temperature, moisture, and light conditions [31]. These environmental gradients interact with other factors, like soil type, geology, and disturbance regimes, to further shape plant distribution patterns. Thus, understanding the combined and singlet effects of these variables could help to design appropriate conservation measures.

The situation is similar in Abraham Sacred forest, which is the portion of the northwestern highlands of Ethiopia, where the area is exposed to anthropogenic pressure and topographically variable. In order to prescribe appropriate conservation actions, this work has been done with the aim of answering key questions (problems) on the effects of terrain variables and disturbances in the status of Abraham Sacred Forest. The objectives of this work were, thus, to a) identify the plant community types; b) analyze the effect of terrain variables, and disturbances in shaping plant community formations and c) evaluate the status of the vegetation structural pattern.

## Materials and methods

### Description of the study area

**Fig 1:**
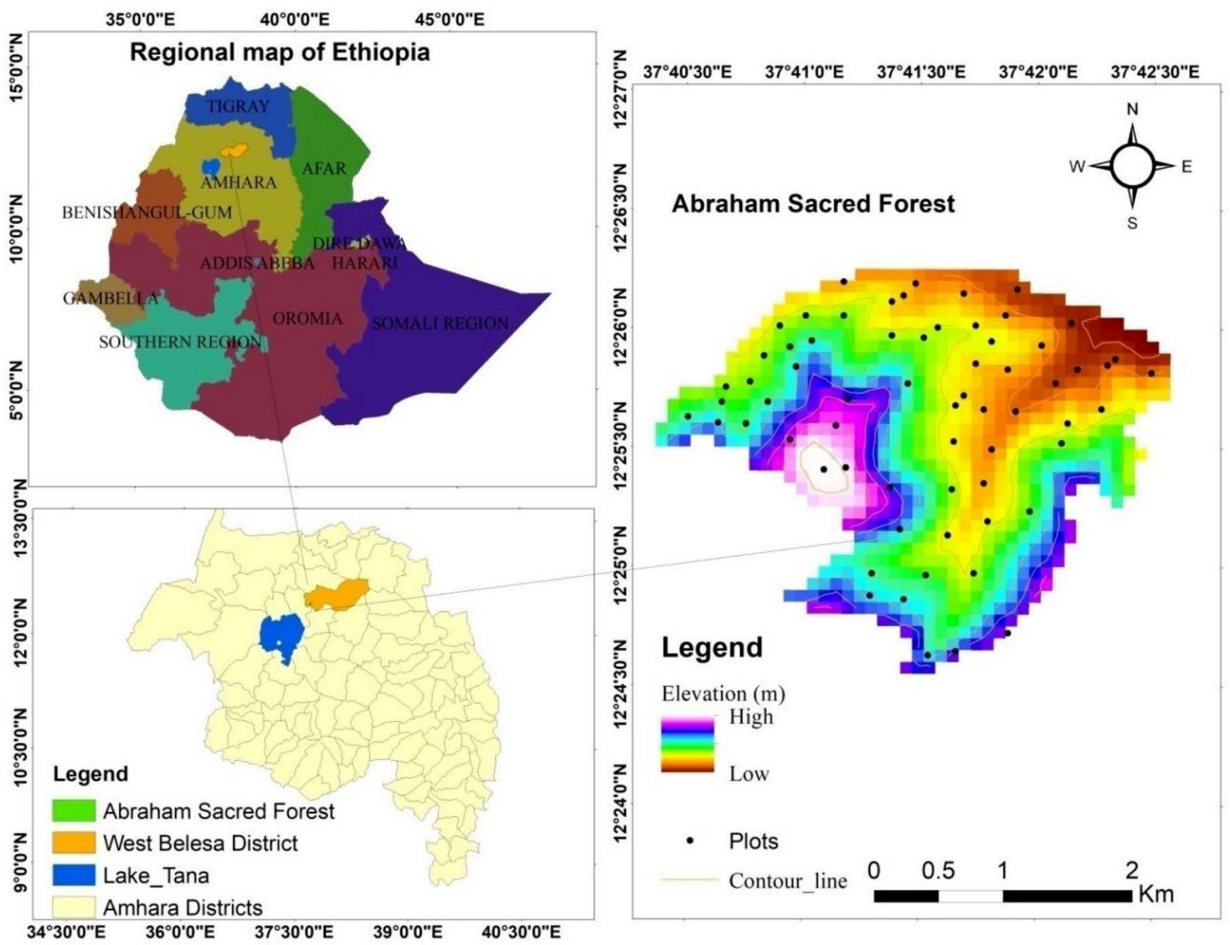
Location map of the study area.

Abraham Sacred Forest is located in West Belessa District, Central Gondar Zone, Amhara National Regional State, northwest Ethiopia. The District is found 860 km northwest of Addis Ababa, the capital of Ethiopia. The altitude of the District ranges from 900 to 2565 meters above sea level and that of the study forest ranges about 1965 to 2335 m. The geographical position of the study forest ranges from 12° 24’ 30” - 12° 26’ 30” N latitude and 37° 40’ 30” - 37° 42’ 30” E longitude with a total area of 546.88 hectares (Fig 1).

The study area consists of chains of rugged mountains, flat and plain areas. Its agro-ecology is predominantly lowland (59.8%), followed by midland (38.7%) and highland (1.5%). The vegetation of the study area is largely dry evergreen afromontane forest and grass land complex with the characteristic species such as *Dodonaea viscosa* subsp. *angustifolia* (L.f) J.G. West, *Damnacanthus indicus* C.F. Gaertn, *Jasminum abyssinicum* Hochst. ex DC., *Oleo europaea* subsp. *cuspidata* (Wall & G. Don) Cif. The analysis of meteorological data from 2015 to 2023 revealed that the study area exhibits a unimodal rainfall pattern. The mean annual rainfall is 911.5 mm per year, with peak rainfall occurring in July and August that gradually fall down from September to May. The mean annual temperature of the study area is about 24.5°C, ranging from a mean minimum of 18.9°C to a mean maximum of 36°C. The hottest months are April and May.

### Sampling design

A reconnaissance survey was conducted in November 2022 in order to have a full picture of the study sites during which the general features of the study area were inspected, sampling sites of the study area were identified, data collection technique and direction for the alignments of the transects were determined. In the present work, data in the whole range of the forest were collected using eight parallel line transects with variable length, spaced at 200 m intervals that were laid across the forests in a north-south direction. A total of 60 sampling plots with a size of 20 m x 20 m were laid at 100 m intervals along the transect lines systematically. Within each of the main plots, five subplots, with the size of 2 m x 2 m were set; one at each corner and one at the center to collect two sets of vegetation data: seedlings and saplings. For the proper organization of the data, woody plant species were classified into three size (age) classes; seedling, sapling and mature plants based on their height and diameter at breast height. For the present work, seedlings were operationally defined as all woody individuals with DBH < 3 cm and height < 1.5 m; adults as all woody plants with DBH ≥ 3 cm and height > 3 m, and seedlings as woody individuals with DBH < 3 m and height > 1.5 m but < 3 m.

### Vegetation and environmental data collection

All the woody plant species (with their individuals) were recorded in each sample plot by their local or scientific names or using codes. To compile the complete list of species in the forest, new plant species occurring out of plots but inside the forest within 10–15 m distance from plots were also recorded however these species were not included in subsequent quantitative data analyses [32]. Based on their life (growth) form, woody plants were classified as trees, usually tall (> 5 m) with single dominant trunk and defined canopy; shrubs, up to five meter height with multiple stems that lack defined canopy and liana with trailing or twining stem. Specimens of woody plant species were collected, coded, and properly dried up using plant presses, and later identified in the Herbarium of University of Gondar. For identification of plant specimens, flora of Ethiopia and Eritrea, Plants of the world online and www.ipni.org were used. The girth of each mature tree and shrub species was measured at 1.3 m above the ground which was later converted into DBH. In each plot, percent cover-abundance data of a woody plant species was recorded and later standardized by converting the value into modified 1–9 Braun-Blanquette scale [33].

Terrain variables such as altitude, aspect, slope and locations were measured and recorded for each sample plot. Altitudes and locations were measured using Garmin GPS 60; slope (in %) and aspect were measured using Suunto Optical Reading Clinometer and compass respectively. Values for aspect were codified based on [34], where N = 0, NE = 1, E = 2, SE = 3, S = 4, SW = 3.3, W = 2.5, NW = 1.3 before analysis. In order to match computation using R-software, the data were, then, organized in an Excel spread sheet into a data matrix of sample plots (row) and species abundance (column). The effects of disturbance intensity was estimated by assessing number of stumps, proportion of bare ground and abundance of faecal droppings by herbivores, and by locating evidence of trampling, browsing and animal trails in each sample plot. Following [35], the intensity levels were, then, designated as “0” for no or slightly grazed, “1” for intermediately grazed, “2” for intensively grazed and “3” over grazed respectively to fit the data with the software program.

### Vegetation data analyses

#### Species area curve and rarefaction

In order to check whether representative data were collected in the forest or not, a species accumulation curve was plotted using R software version 4.3.3. In addition, nonparametric species richness estimators including Abundance coverage estimate (ACE), Chao1, Chao 2, Jackknife 1, Jackknife 2 and Bootstrap were used to estimate the number of species in the forest. These non-parametric species richness estimators are applicable to quadrat-based data sets that can be treated as either the random samples of space or as fixed samples of individuals [36]. Moreover, these estimators are free of parametric (normal) species abundance distribution model [37]. By employing appropriate formulae of the different nonparametric estimators, species richness estimation was computed using EstimateS version 9.1. In order to calculate the mean estimator and expected number of species for each sample accumulation level, the sample order was randomized 100 times.

#### Plant community determination

Hierarchical cluster analysis was performed using R software version 4.3.3 to classify the vegetation into plant community types. Sixty sample plots and 70 species abundance data were subjected to cluster analysis. Similarity ratio was used to determine the resemblance function and Ward’s method to minimize the total within group mean square or residual squares [38]. The optimal numbers of clusters were determined objectively by Elbow method where it plots the number of clusters with the corresponding sum of the square distances within clusters. The plot resembles an elbow and the optimal cluster is located at the sharp bend (elbow) [39]. The community types identified from the cluster analysis were further refined in a synoptic table and species occurrences are summarized as a synoptic-cover abundance values. Synoptic values are the product of the species frequency and average cover abundance values [33]. The dominant species that were used to name each plant community were identified based on their higher synoptic values.

After subjecting the floristic data to the Shapiro-Wilk normality test, it became evident that the data exhibited a skewed distribution (p-value < 0.05), suggesting that the data were not normally distributed. As a result, the Kruskal-Wallis test, a non-parametric statistical method, was used to examine the significance of environmental variables in explaining the patterns of plant community formation [39]. In order to check whether the variation among plant community types was significant or not with regard to non collinear significant environmental variables, Tukey’s pairwise comparison test was computed using R software version 4.3.3. Tukey’s honestly significant difference (Tukey’s HSD) test was used to find means of environmental variables that are significantly different between two communities at a time, i.e. it applies simultaneously to the set of all pair-wise comparisons and identifies any difference between two means that is greater than the expected standard error [39].

#### Plant species diversity analysis

In order to compare species diversity and evenness among community types, Shannon-Wiener Diversity Index [17] was computed by using package vegan in R software version 3.5.1

$$H'=-\sum_{i=1}^{S}\;p_{i}\;\text{ln}\;p_{i}$$

$E=\frac{H'}{H{'}_{\max}}=-\frac{\sum_{i=1}^{S}\;p_{i}\;\text{ln}\;p_{i}}{\text{ln}\;s}$[40], where H’ = the value of the Shannon-Weiner diversity index, s = the number of species in the community, Pi = the proportion of individuals of the i^th^ species expressed as a proportion of total cover, ln = log base, J = Evenness of species in the sampling area, H ‘max = maximum value of diversity.

#### Species similarity analysis

So as to compare species composition similarity among community types, Sorensen’s similarity coefficient was calculated by the formula Ss = 2a/ (2a+b+c) [40], where Ss = Sorensen’s similarity coefficient, a = number of species common to both communities, b = number of species present in one community but not in other and c = number of species present in other community.

#### Vegetation structure data analyses

Frequency, density, DBH, basal area and importance value index (IVI) were used for the analyses of vegetation structure. Frequency (F) calculated as a total number of plots in which the species occur/total number of plots studied x 100. Based on Raunkiaer’s" law of frequency [41], the frequency of the forest species were sorted in increasing order and grouped into 5 classes (in %): frequency class 1(1–20), 2(20.1–40), 3(40.1–60), 4(60.1–80) and 5(80–100). The percentage distribution of species in each class was used to assess the distributional patterns of species in the forest. Relative frequency (RF) of a species = frequency of the species in stand/sum of the frequencies of all species in the stand x 100. Density (D) was defined as the number of individual plants of a species per unit area sampled [40]. D = number of above ground stems of a species/Sample area in hectare x 100. The relative density (RD) of the species is calculated as the density of a species /total density of all species x 100, where D is expressed as a percentage for each species and then sorted in increasing order and grouped into eight density classes (in stems ha^-1^) as density classes 1 (<30), 2 (30.1–60), 3 (30.1–90), 4 (90.1–120), 5 (120.1–150), 6 (150.1–180), 7 (180.1–210), and 8 (>210).

Diameter at Breast Height (DBH) was calculated from girth of each adult woody species using the formula DBH = C/π, where DBH = diameter at breast height, C = circumference, and π = constant with a value 3.14. The diameter at breast height was classified into five DBH classes (cm). The percentage number of individuals in each DBH class was calculated so as to assess the structural patterns of the forest species. Basal area was calculated based on the value of DBH on the uphill side of the tree by using the Formula BA = π d2/4 [40], where BA = Basal area in m^2^ per hectare, d = diameter at breast height in meter. π = 3.14. Basal area of the forest species were sorted in ascending order and were grouped in to basal area classes which was used to assess the structural pattern of the vegetation. Importance Value Index (IVI) was calculated from the sum of relative frequency (RF), relative density (RD) and relative dominance (RDo) [40], IVI = RF+ RD+ RDo). Dominance is measured in term of basal area where relative dominance (RDo) is the proportion of the basal area of a species to the sum of the basal coverage of all the species in the area.

#### Regeneration status data analysis

Regeneration status of tree and shrub species was analyzed using density ratios between age classes (ratios of seedlings to saplings and adults; ratios of saplings to adults). Based on [42] and [43] regeneration status of forests are classified as follows

1. “Good“ regeneration, if seedlings > saplings > adults;
2. ”Fair” regeneration, if seedlings > or ≤ saplings ≤ adults;
3. “Poor” regeneration, if the species survives only in sapling stage, but no seedlings (saplings may be <, > or = adults);
4. “None” if a species is present only in an adult form but a species is absent both in sapling and seedling stages and
5. “New” if a species has no mature, but only sapling or seedling stages

## Results and discussion

### Floristic composition

**Fig 2:**
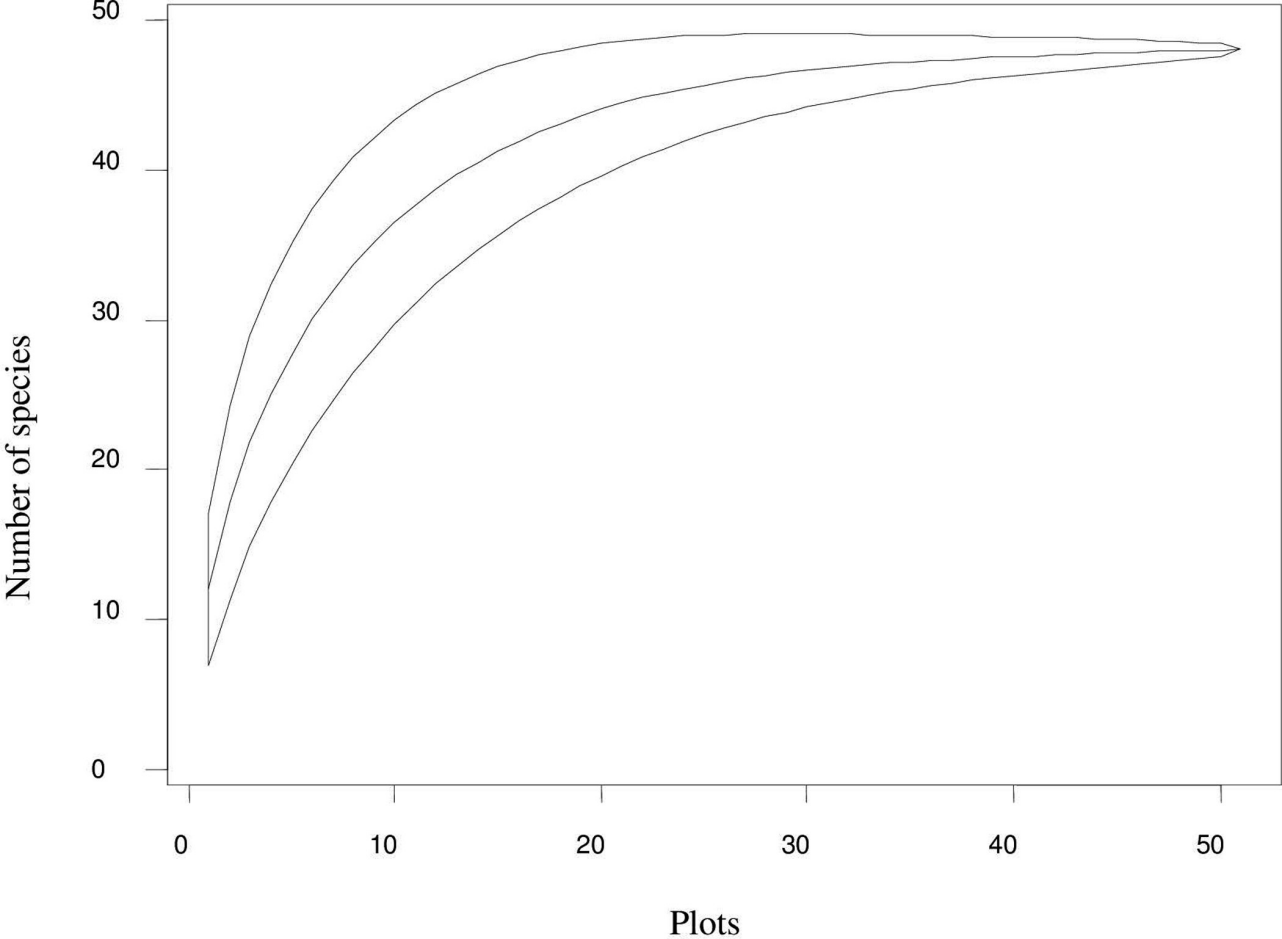
Species accumulation curve.

In order to affirm the representativeness of data collected in the forest, Species Accumulation Curve was plotted. The curve shows whether the species in the study forest are exhaustively surveyed or not that would eventually reveal the real floristic composition of the study area. A curve labeled off (or in other case a curve approximately reaching an asymptote) indicated that no new species would be collected if sampling effort is further continued. The Species Accumulation Curve in adequately sampled study area levels off (asymptote) before the total number of sampling plots is reached as demonstrated in Fig 2 [39]. This could prove that the study area is exhaustively sampled.

In addition, the species richness of the Abraham Sacred Forest patch was estimated using different estimators (Table 1). All the species richness estimators showed more or less similar species richness to the observed number of species (Sobs). Though more or less similar, relatively the highest species richness estimation (70.9) was obtained by Chao2 estimator (Table 1). Based on these different estimators, most species of the study area (96.3–99%) were collected proving the representativeness of the collected data.

**Table 1:** Number of samples, observed species (Sobs) and estimated species richness based on different estimators of the forest patch.

Characteristics	Study Forest
No of samples	60
Sobs	69.9
ACE	70.3
Chao1	70.7
Chao2	70.9
Jacknife1	70.1
Jacknife2	70.0
Bootstrap	70.11
Species collection degree	96.3–99%

A total of 70 woody species (trees, shrubs and woody climbers) representing 62 genera and 38 families were identified in the study forest (S1 Table). The study forest’s (size = 546.8 ha) species richness (70 species) was smaller than other dry evergreen afromontane forest patches studied in a similar manner such as [44] in Tara Gedam Monastery (size = 975 ha) with 111 species, [45] in Gond Teklehaymanot Monastery forest (size = 800 ha) with 80 species, but higher than [46] in Yemrehane Kirstos Monastery Forest (size = 200 ha) with 39 species and [47] in Wanzaye (size = 274 ha) with 49 species. After investigating 28 sacred church forests in South Gondar Zone, Ethiopia, [18] reported 15 to 78 species (37.5 species on average). In addition, [48], after reviewing 53 Ethiopian Orthodox Church sacred forests, reported species richness ranging from 16 to 200 (57.8 species on average). Thus, the species richness of the current study forest (70 species) was found within the range of sacred church forests studied in Ethiopia before.

However, authors who studied sacred forests outside Ethiopia reported much higher species richness than the current study. In East Africa, [49] who studied different sacred sites (including Mount Kenya, Ramogi Hill, Loita Forest, Nzaul Hill,) disclosed species richness counted in hundreds. [50] who investigated in Boixangshan Holy Hill, Manlang Holy Hill, Manyangguag Holy Hill, Mengla and Menglen Natural Reserves of China sacred forests declared 144, 90, 142, 207 and 332 species richness, respectively. These reports demonstrated that different forest patches support varying numbers of species. These differences in species richness among various forest patches might be attributed to environmental variables that create habitat heterogeneity with several microhabitats that favor different species survival [51] and the climate of the area that could shape the vegetation nature and disturbance level [52]. Other factors such as conservation status, geographical location, size of the forest and successional stage, might also cause differences in species richness.

Investigating sacred forests like the present study, provide fundamental scientific information regarding the conservation status and values of these vital resources as well as fills the knowledge gap in the filled of ecology. In Ethiopia, especially in the northern part, forests are found in fragmented patches, of which sacred forests are the most pertinent because they represent remnants of natural ecosystems in extensively deforested areas. These numerous forest patches have several cultural and ecological values [18]. Culturally, they are often regarded as living museums, where ancient customs, beliefs, and oral traditions are preserved and passed down through generations [19]. Ecologically, they serve as biodiversity hotspots that support a wide array of plants and animals including endemic, rare and endanger species [18, 53]. They are also the sources of genetic diversity maintaining the resilience of ecosystems, and other ecosystem services. In addition, they serve as corridors, connecting fragmented landscapes that facilitate the movement of wildlife, genetic exchange, and overall ecosystem health [19].

**Fig 3:**
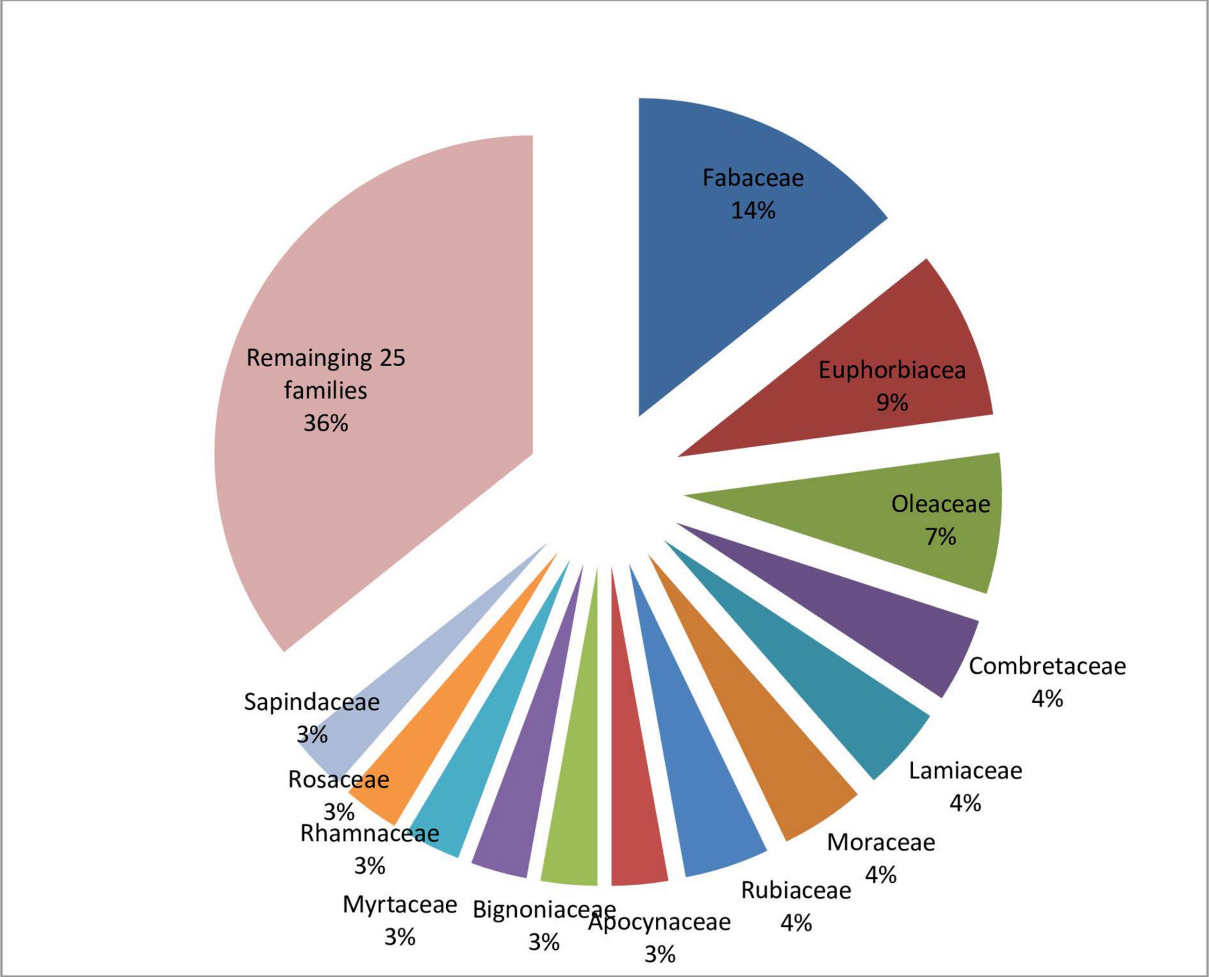
Percent species contribution of families.

Regarding growth form, the forest was composed of 53.7% trees, 35.8% shrubs and 10.4% lianas. Similar result was observed in other Ethiopian Church Forests [19], Yemrehane Kirstos Monastery Forest [46] and Gond Teklehaymanot Monastery forest [45]. In relative terms, tree species are less tolerant to disturbance compared to shrubs and tend to appear in the later stages of ecological succession. This would suggest that in sacred forests, where disturbance is minimal, the forest patches are able to support a higher proportion of tree species. Therefore, the dominance of trees is a characteristic feature of old age sacred forests conserved for hundreds of years allowing orderly ecological succession during which other growth habits were largely replaced by trees. Fabaceae was the most dominant family with 10 species (14%) (Fig 3). The dominance of Fabaceae was also recorded in the Flora of Ethiopia and Eritrea (second dominant next to Asteraceae), Ethiopian Church Forests with 17 species [19], Tara Gedam Monastry with 17 species [44] and Waldiba Monastery forest represented by 13 species [54]. From these reports it can be noted that the species of Fabaceae appear to be well-established across the different sacred forest patches in Ethiopia. The dominance of this family might be attributed to its evolutionary adjustment (adaptation) to a variety of ecological circumstances. The species ability to form symbiosis help them to thrive in nitrogen-deficient soils; their ability to form extensive root system enable them to capture nutrients and water from the soil by outcompeting other species and some Fabaceae species releases chemicals (allelopathy) that suppress the growth of neighboring plants giving a competitive advantage for Fabaceae species [55, 56].

### Woody species diversity, richness and evenness

The Shannon-Wiener diversity index for the woody species in the entire forest was 3.57, and the evenness was 0.92. Shannon-Weiner diversity value is high when it is above 3.0; medium when it is between 2.0 and 3.0 and low when it is smaller than 1.0 [40]. Thus, the species diversity of the Forest was high and greater than similar Monastery Forests such as Yemrehane Kirstos (H’ = 2.88) [46], Abbo Sacred Forest (H’ = 2.99) [57] and Zijje Maryam (H’ = 3.29) [58]. These reports indicated that the sacred areas in Ethiopia support forests with varying levels of species diversity. In addition, compared to the observed total species of Abbo Sacred Forest (J = 0.75) [57], Tara Gedam Monastery forest (J = 0.65) [44] and Gosh Beret (J = 0.66) [59], Abraham Sacred forest had higher overall evenness (J = 0.92) indicating the presence of a relatively much more equitable distribution of individuals among various species. A forest with high species diversity and evenness would have proper interaction among species that contribute for the better ecosystem health and stability.

### Plant community types

**Fig 4:**
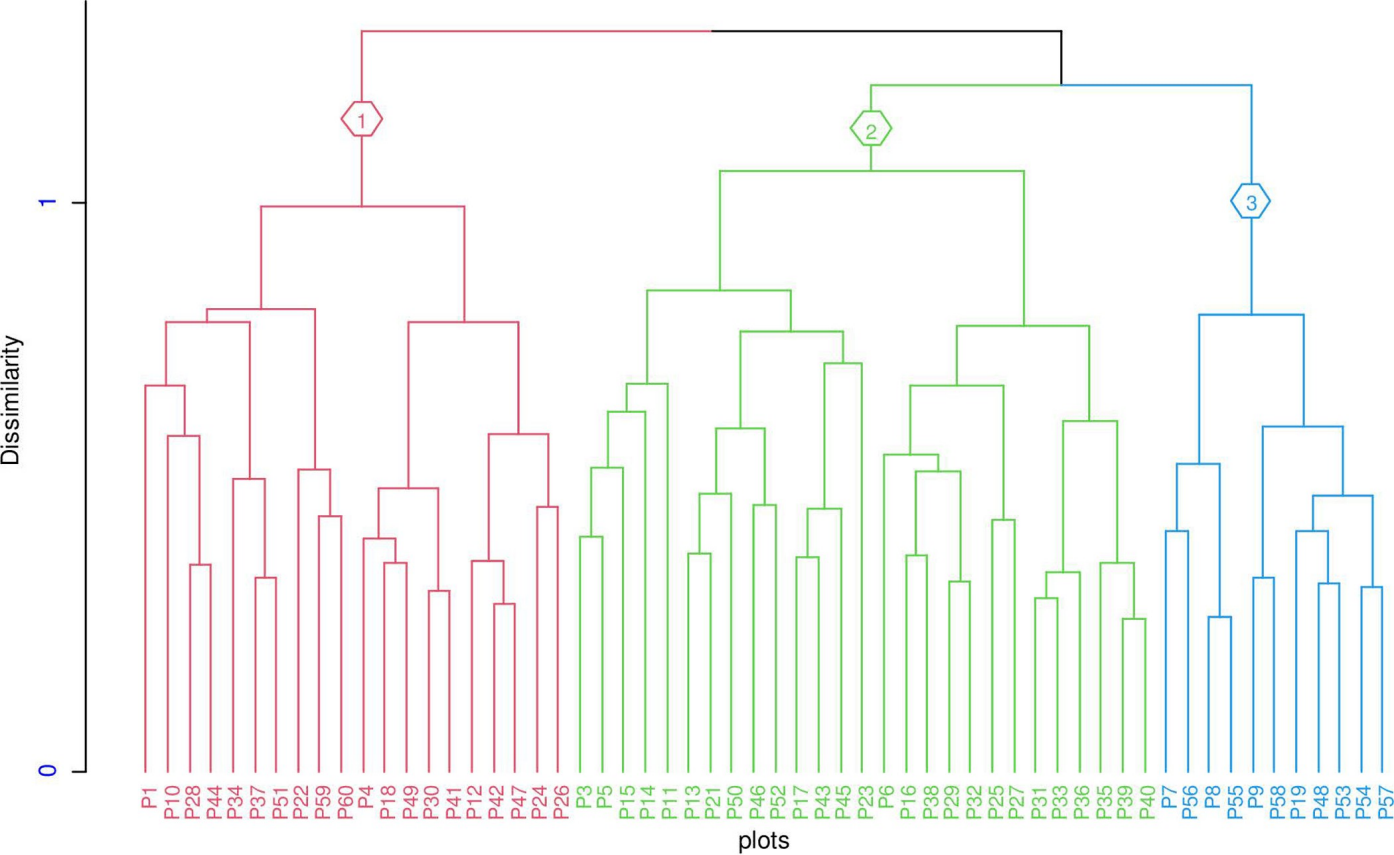
Clusters generated from hierarchical cluster analysis.

Three different plant communities were identified from the cluster analysis of the floristic data (Fig 4). The communities were described based on their synoptic cover value. Species with higher synoptic cover-abundance value [33] are considered as dominant species and characteristic species were those with high frequency in the type and a lower frequency in most other clusters. Based on this principle, the community types were briefly described below. The three communities were found to have different species diversities ranging from 3.71 to 3.94 with a mean value of 3.8 and richness values ranging 54 t0 59 with a mean value of 57.

- *Premna schimperi* Engl.- *Olea europaea* subsp. *cuspidata* community type (C1)
  The species within this community type were predominantly found at higher altitudes, ranging from 2255 to 2289 m.a.s.l. The community was represented by 22 plots in which 54 species were clustered. A significant proportion of the plots (22.7%) were situated on west and east facing slopes each. The majority of plots were distributed in areas characterized by relatively steep slopes or sloppy terrain. The community exhibited the lowest level of species diversity (3.71), species evenness (0.89), and species richness (54) (Table 2). One of the reasons for the lowest species diversity and richness was that most of the plots were located near the forest margin, where agricultural expansion and other anthropogenic activities had highly encroached. In addition, the sloping nature of the area contributes to soil erosion, which can lead to reduced soil thickness, lower soil moisture levels, and decreased nutrient content. Along with the dominant species used to name the community, *Senegalia polyacantha* (Willd.) Seigler & Ebinger, *Prunus africana* (Hook.f) Kalk. *Searsia glutinosa* (Hochst. ex. A. Rich.) Moff., *Olea capensis* L., and *Gymnanthemum amygdalinum (Del*.*) Sch-Bip*. *ex Walp*. *Premna schimperi*, *Osyris laceolata* Hochst & Steud., *Rydingia integrifolia* (Benth.) Sch. &V.A. Alb. and *Rumex nervosus* Vahl were the common woody species in the community (Table 3).
- *Acokanthera schimperi- Vachellia abyssinica* community type (C2)
  This community type was distributed between the altitudinal ranges of 2247–2273 m.a.s.l. It was represented by substantial number of plots (27) with a total of 55 associated species (Table). Over 25% of the plots were oriented towards southeast-facing slopes, while around 18% of the plots faced northwest. The majority of plots (41%) were distributed in areas characterized by relatively gentle slopes. This community exhibited 3.74 species diversity and with 0.89 evenness. It was the second in richness (55) and diversity, next to the community three (Table 2). The observed pattern could be attributed to the plots’ considerable distance from the village, and the fact that most of them were situated on gentle to medium slopes with comparatively low soil erosion. In addition, due to the local belief that this particular part of the forest serves as a habitat for wild animals, including hyenas, there is a reduced influence of anthropogenic activities (disturbance) compared to other communities [59].
  *Combretum pisoniiflorum* (Klotz.) Engl., *Ekebergia capensis* Sparrm., *Vachellia* abyssinica (Hochst. ex Benth) Kyal. & Boatwr., *Croton macrostachyus* Hochst. ex Del., *Acacia hamiltoniana* Maiden, *Terminalia brownii* Fresen. and *Ficus thonningii* Blume were tree species dominated the community. *Acokanthera schimperi* (A.DC.) Benth & Hook f. ex. Sch., *Premna schimperi*, *Ormocarpum pubescens* (Hochst) Cufod. ex J.B. Gillett and *Rydingia integrifolia* were preferably distributed shrub species. *Euclea racemosa L*. *Dodonaea viscosa* subsp. *angustifolia*, *Dichrostachys cinerea* (L.) Wight & Arn., and *Damnacanthus indicus* var. *indicus* were shrubs commonly abundant in all the three communities. *Rosa abyssinica* R.Br. and *Pterolobium stellatum* (Forssk.) Bren. were very common liana species found in all the communities (Table 3).
- *Cordia africana* Lam.*- Capparis tomentosa* Lam. community type (C3)
  Species in this community type were distributed in the lower altitudes ranging from 2243–2265 m.a.s.l. It was represented by 11 plots and 59 associated species (Table 2). Plots were largely distributed in gentle (medium level) slopes. The species in these plots did not exhibit any particular preference for slope facing. Though the community was represented by the smaller number of plots (11), this community exhibited the highest level of diversity (3.94), with the maximum species richness (59) and evenness (0.93) (Table 2). The flat nature or gentle slope of the site, which contributes to reduced soil erosion and better availability of soil moisture and nutrients, along with the greater distance of plots from anthropogenic disturbances contribute to the highest observed species diversity. This community was predominately characterized by the presence of tree species. Among others, *Allophylus abyssinicus* Radlk, *Croton macrostachyus*, *Vachellia abyssinica*, *Combretum pisoniiflorum*, *Ficus thonningii*, *Adansonia digitata* L., *Gardenia ternifolia* Schumach. & Thonn, *Ficus sur* Forssk. and *Erythrina abyssinica* DC. were commonly available trees under this community. *Gymnosporia arbutifolia* (Hochst. ex A. Rich.) Loes., *Justicia schimperiana* (Hochst. ex Nees) T. Anderson, *Brucea antidysenterica* J.F. Mill., *Rotheca myricoides* (Hochst.) Ste. & Mabb. and *Pavetta abyssinica* Fresen. were frequently occurred shrub species. A significant number of liana species, such as *Jasminum grandiflorum* L., *Helinus mystacinus* E. Mey & ex. Steud., *Cissus populnea* Guill & Perr., and *Phytolacca dodecandra L’Hér*., were found to be preferentially distributed within this community (Table 3). The richness of liana species in this community may be attributed to the presence of trees that provide suitable support for their climbing habit.

**Table 2:** The plots, altitudes, species richness (S), diversity (H') and evenness (J) of each community types.

Cluster	Altitude range	Plots No	Plots	S	H'	J
1	2255–2289	22	1,2,4,10,12, 18, 20, 22,24, 26, 28,3034,37,41, 42, 44,47,49, 51,59, 60	54	3.71	0.89
2	2247–2273	27	3,5,6,11, 13–17, 21, 23, 25, 27, 29, 31–33, 35, 36, 38–40, 43, 45, 46, 50, 52	55	3.74	0.89
3	2243–2265	11	7–9,19, 48, 53–58	59	3.94	0.93
			Mean ±Sd	56 ±2.65	3.8±0.13	0.9±0.02

**Table 3:** Species with a synoptic value ≥ 1.5 in at list one community (dominant species are indicated in bold).

No	Scientific name	Cluster 1	Cluster 2	Cluster 3
1	*Acacia hamiltoniana*	0.36	1.78	0.36
2	*Acokanthera schimperi*	2.27	**4.48**	2.27
3	*Adansonia digitata*	0.82	0.33	1.91
4	*Allophylus abyssinicus*	2.68	2.70	3.73
5	*Brucea antidysenterica*	0.36	0.33	2.18
6	*Calotropis gigantea var*. *procera* (L.) Dryand	0.41	0.11	1.64
7	*Calpurnia aurea* Brum.	3.32	3.41	4.18
8	*Capparis tomentosa*	1.09	0.44	**3.91**
9	*Cissus populnea*	0.45	0.22	2.91
10	*Combretum adenogonium* Steud. ex A. Rich.	1.00	1.33	1.64
11	*Combretum pisoniiflorum*	0.36	2.41	2.45
12	*Cordia africana*	1.32	0.44	**4.00**
13	*Croton macrostachyus*	0.55	2.78	2.91
14	*Damnacanthus indicus* var. *indicus*	4.14	3.89	4.27
15	*Dichrostachys cinera*	4.59	4.70	4.73
16	*Dodonaea viscosa* subsp. *angustifolia*	4.68	4.48	4.82
17	*Dombeya torrida*	0.55	0.56	1.09
18	*Ekebergia capensis*	0.41	2.19	2.36
19	*Erica arborea* L.	0.14	0.56	1.09
20	*Erythrina abyssinica*	0.36	0.81	1.36
21	*Euclea rasemosa*	4.68	4.93	5.00
22	*Ficus sur*	0.14	0.44	1.64
23	*Ficus thonningii*	0.91	1.44	2.27
24	Ficus vesta Foressk.	0.00	0.56	1.55
25	*Gardenia ternifolia*	0.55	0.11	1.64
26	*Grewia ferruginea* Hochst. ex A. Rich.	3.00	4.19	4.55
27	*Gymnanthemum amygdalinum*	1.36	0.33	1.09
28	*Gymnosporia arbutifolia*	0.45	1.11	2.82
29	*Helinus mystacinus*	0.41	0.22	2.18
30	*Heteromorpha arborescens* Cham. & Schltd.	0.27	0.78	1.64
31	*Jasminum grandiflorum*	0.73	0.74	2.55
32	*Juniperus procera* Hochst ex. Endl.	0.27	0.11	1.36
33	*Justicia schimperiana*	1.00	1.04	2.55
34	*Millettia ferruginea* (Hochst.)Hochst. ex Baker.	1.09	0.33	1.36
35	*Olea capensis*	1.64	1.04	0.64
36	*Olea europaea* subsp. *cuspidata*	**2.95**	1.19	1.27
37	*Opuntia ficus- barbarica* (L.) Mill.	0.27	1.22	0.27
38	*Ormocarpum pubescens*	0.68	1.44	0.55
39	*Osyris lanceolata*	1.91	0.59	0.36
40	*Pavetta abyssinica*	0.82	0.78	1.36
41	*Phytolacca dodecandra*	0.27	0.89	1.09
42	*Premna schimperi*	**3.82**	1.59	1.45
43	*Prunus africana*	2.14	0.59	0.36
44	*Psydrax schimperiana* subsp. *accidentalis* Bridson	0.18	1.22	0.73
45	*Pterolobium stellatum*	4.05	4.33	4.73
46	*Rosa abyssincia*	0.91	1.33	0.27
47	*Rotheca myricoides*	1.09	1.15	0.00
48	*Rotheca myricoides*	0.14	0.44	1.91
49	*Rumex nervosus*	1.27	0.59	1.09
50	*Rydinia integrifolia*	1.27	1.33	0.73
51	*Searsia glutinosa*	2.09	0.44	0.73
52	*Senegalia polyacantha*	2.64	0.74	1.09
53	*Terminalia brownii*	0.32	1.52	0.64
54	*Vachelia seyal*	1.14	1.48	1.45
55	*Vachellia abyssinica*	0.36	**2.97**	2.00

#### Species composition similarity among communities

The overall similarity coefficient among communities ranges from 0.48–0.56 (Table 4). The result demonstrated that species composition varies considerably across the community types. The highest similarity (least dissimilarity) was observed between communities 2 and 3 (56%) which might be due to the close proximity of the communities or exposure to similar environmental factors leading them to have similar adaptations. The least similarity was observed between communities 1 and 2 (48%). This might be due to farther distance between the communities that lead to exposure to different environmental factors.

**Table 4:** Sorensen’s similarity coefficient among communities.

Community	C1	C2	C3
C 1	1		
C 2	0.48	1	
C 3	0.49	0.56	1

C1 = community 1, C2 = community 2 and C3 community 3

#### Relationships between community types and environmental variables

Data for altitude, slope, aspect and disturbance were used to show the effect of environmental variables on the patterns of plant community formation. Shapiro-Wilk normality test result (W = 0.9587, p-value < 0.00599) indicated that the data exhibited a skewed distribution (p-value < 0.05). Based on the results obtained from the Kruskal-Wallis test, the three plant communities differed significantly from each other with respect to altitude (x^2^ = 29.712, p < 0.001) and slope (x^2^ = 34.905, p < 0.001), (Table 5). Tukey’s honestly significant difference (Tukey’s HSD) test results also revealed that plant community types differed significantly from each other with respect to altitude and slope (Table 6). Community types 2 and 1, 3 and 1, differed significantly with respect to altitude, and community types 2 and 1, 3 and 2 differed significantly with slope (p<0.001).

**Table 5:** Kruskal-Wallis test.

Factor	x^2^	Df	P ‐ value
Altitude	29.712	2	3.532e-07***
Slope	34.905	2	2.634e-08***
Aspect	0.123	2	0.940 4
Disturbance	6.933	2	0.05123

x^2^ = chi-square, Df = degree of freedom, Pr = p- value

**Table 6:** Tukey’s pairwise comparisons of communities based on altitude and grazing (Diff = Difference, lwr = lower, upr = upper, p adj = probability adjusted or p- value).

**Altitude**				
**Community**	**diff**	**lwr**	**upr**	**p adj**
2–1	-20.196970	-28.43010	-11.963836	0.0000006***
3–1	-29.045455	-39.63091	-18.459997	0.0000000***
3–2	-8.848485	-19.10203	1.405057	0.1037734
**Slope**				
**Community**	**diff**	**lwr**	**upr**	**p adj**
2–1	-3.550505	-4.414514	-2.68649609	0.0000000
3–1	-1.136364	-2.247232	-0.02549497	0.0538826
3–2	2.414141	1.338105	3.49017792	0.0000040

Signif. codes: * = p ≤ 0.05, ** = p ≤ 0.01, ** = p ≤ 0.001

**Fig 5:**
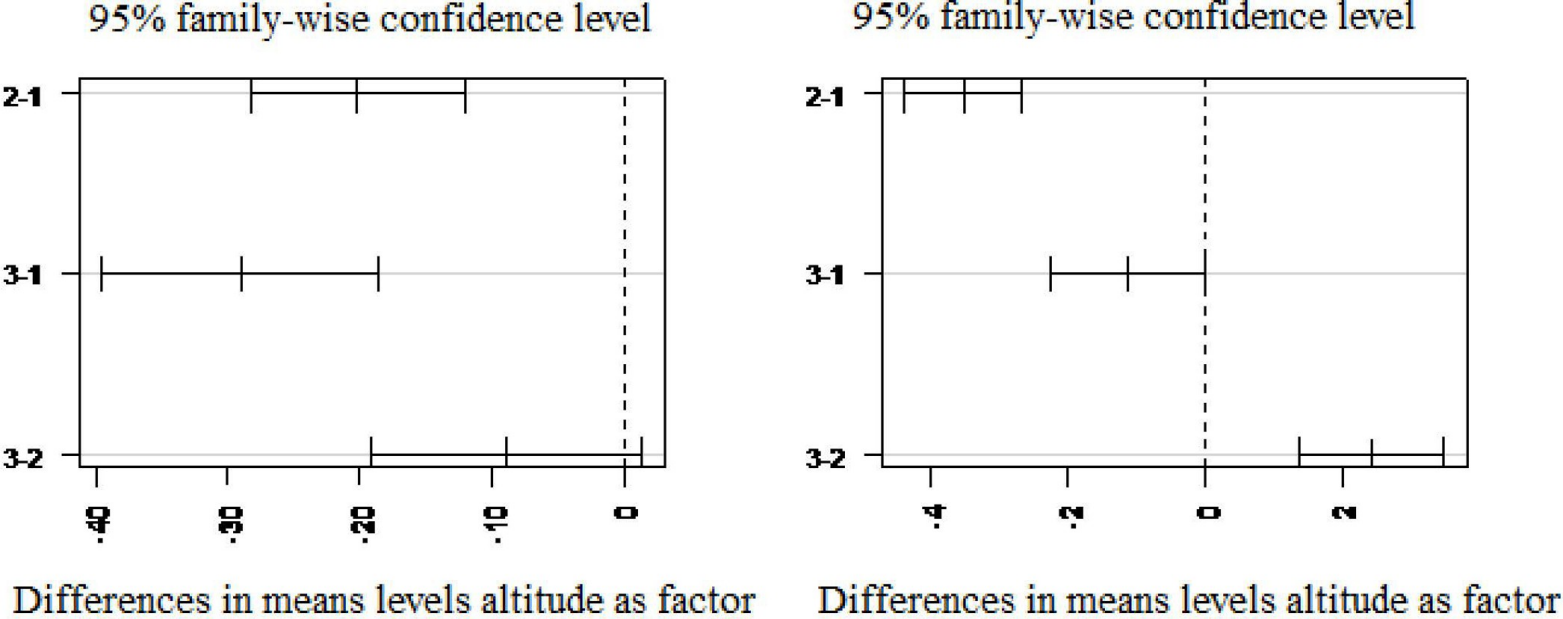
Effect of altitude and slope on plant community formation.

**Fig 6:**
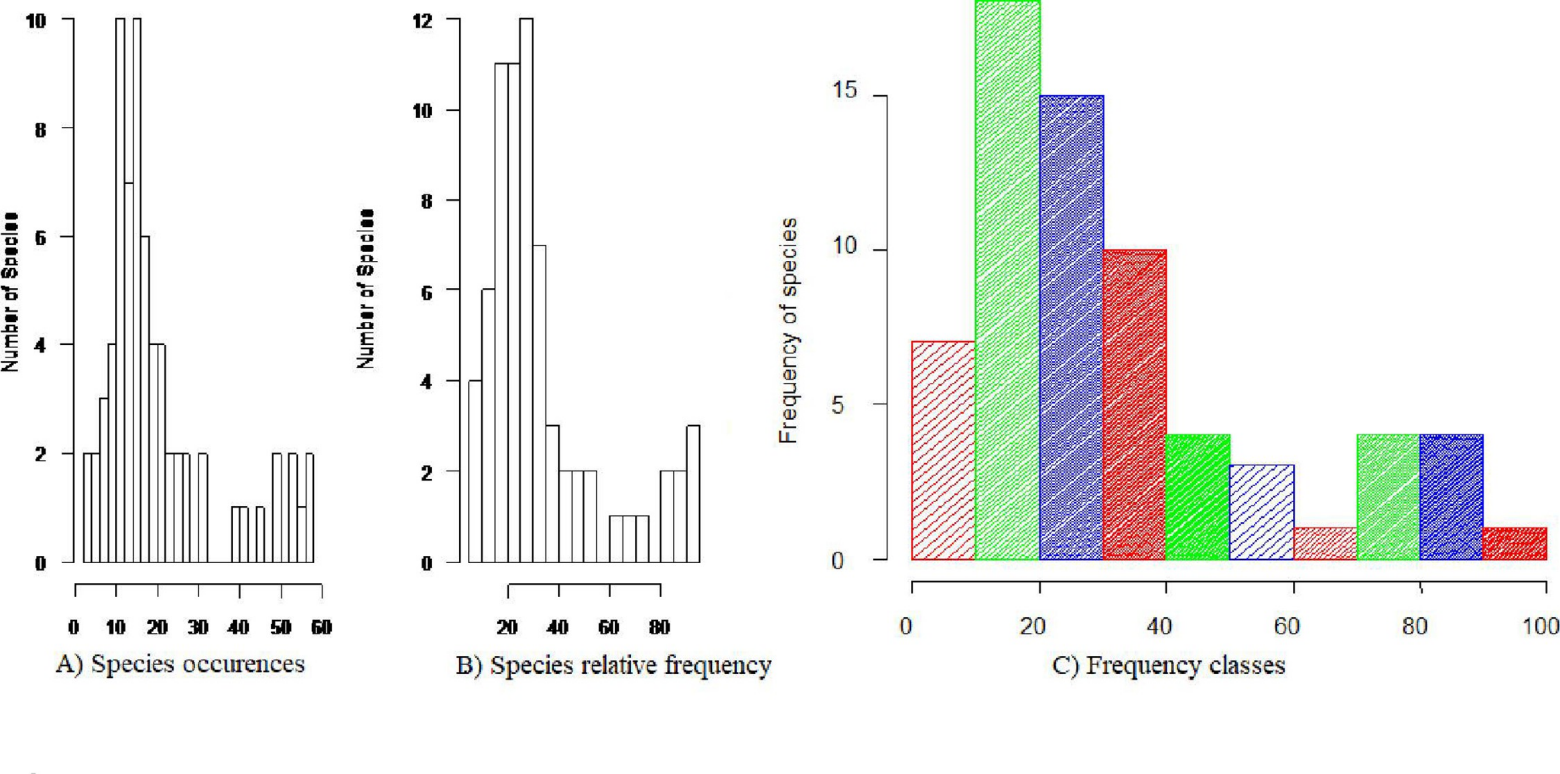
Distribution of species across frequency classes.

The significances of the two variables were further verified using the graphical representation (Fig 6). Graphical representation of the output indicated that comparisons where the mean difference confidence interval does not span (cross) zero were statistically significant in these groups. Thus, communities 2–1 were significantly differentiated by both altitude and slope. In addition, community types 3–1 and 3–2 were significantly segregated in to different units due to the influence of altitude and slope, respectively (Fig 5). Thus, the result indicated that terrain variables were among the factors responsible for the formation of different community types across the forest.

Although there are overlaps among community types in their elevation range, altitudinal variation among community types was one of the variables that segregate the vegetation into plant community types. Several authors [27, 60–63] have used altitude as a proxy or indirect environmental variable that co-varies with other direct environmental variables such as climatic factors (e.g. rainfall, temperature, etc.) and edaphic conditions which influence community formation, growth and distribution directly. In addition, authors [60, 64, 65] pointed out that slope strongly brings variation in species distribution and plant community formation by affecting other environmental variables. Variation in slope has a strong influence on soil thickness, moisture content and chemical properties of soil. Soil on steeper slopes is influenced by bedrock, and tends to be less moist and less acidic [66] that leads to support different plant species from gentle slopes. Some other environmental factors that are not included under this study are also believed to play roles in the formation of plant community types.

### Vegetation structure of the forest

#### Frequency

The species with most frequent occurrence were *Dichrostachys cinera* (93.3%), *Euclea rasemosa* (88.33%), *Dodonaea viscosa* subsp. *angustifolia* (86.67%), *Pterolobium stellatum* (86.67%) *Acokanthera schimperi* (83.3%), *Damnacanthus indicus* var. *indicus* (78.34%), *Grewia ferruginea* (76.6%) and *Calpurnia aurea (*73%) (S1 Table), respectively. Frequent occurrences indicate regular horizontal distribution of the species in the forest that revealed successful reproduction and adaptation.

However, further analysis of frequency data, using figures of species occurrence, relative frequency and frequency classes, revealed that higher number of species were frequently occurred in the lower frequency classes (Fig 6), i.e. most of the species have lower occurrences with lower relative frequency largely distributed in lower frequency classes (frequency classes 1 and 2). This implies that large number of species did not have regular horizontal distribution or might have uneven distribution (or rare occurrences) across the forest. Frequency describes the homogeneity and heterogeneity of a given vegetation, with higher homogeneity being indicated by a higher proportion of species in higher frequency classes and a lower proportion of species in lower frequency classes, whereas higher heterogeneity is achieved by a higher proportion of species in lower frequency classes and a lower proportion of species in higher frequency classes [67]. Thus, the result of this work proved the heterogeneity of the vegetation. This would imply that several species are not successful in reproduction and adaptation.

#### Density

The total density of woody species (DBH ≥ 3 cm) in Abraham Monastery Forest was 4580.4 trunks ha^-1^. Previous authors in similar Monastery forests such as [19] in South Gondar Zone Monastery forests (2707 stem ha^-1^ in Emashenkor, 1725 stem ha^-1^ in Wonkesht, 1634 stem ha^-1^ in Amstya and 1324 stem ha^-1^ in Gunaguna), [44] in Tara Gedam Monastery forest (3001 stem ha^-1^), [68] in Debre Libanos Monastery forest (459.5 stem ha^-1^) and [54] in Waldiba Monastry forest (1906.6 stem ha^-1^) reported lower density. In one of his study of Monastery forests (Mekedese Mariam), [18] disclosed comparable density (i.e. 4644 stem ha^-1^) to the density of Abraham Sacred forest. The results from the various studies showed that sacred forests contain varying total density. The variation in density of species across different forests could be attributed to differences in managerial interventions where in strictly protected Monastery forests, effects of trampling and grazing on seedling and sapling density decrease. Differences in topographic features (habitat preference), human pressure and age or successional stage of the Monastery forest might also bring variation in the density of the monastery forests.

**Fig 7:**
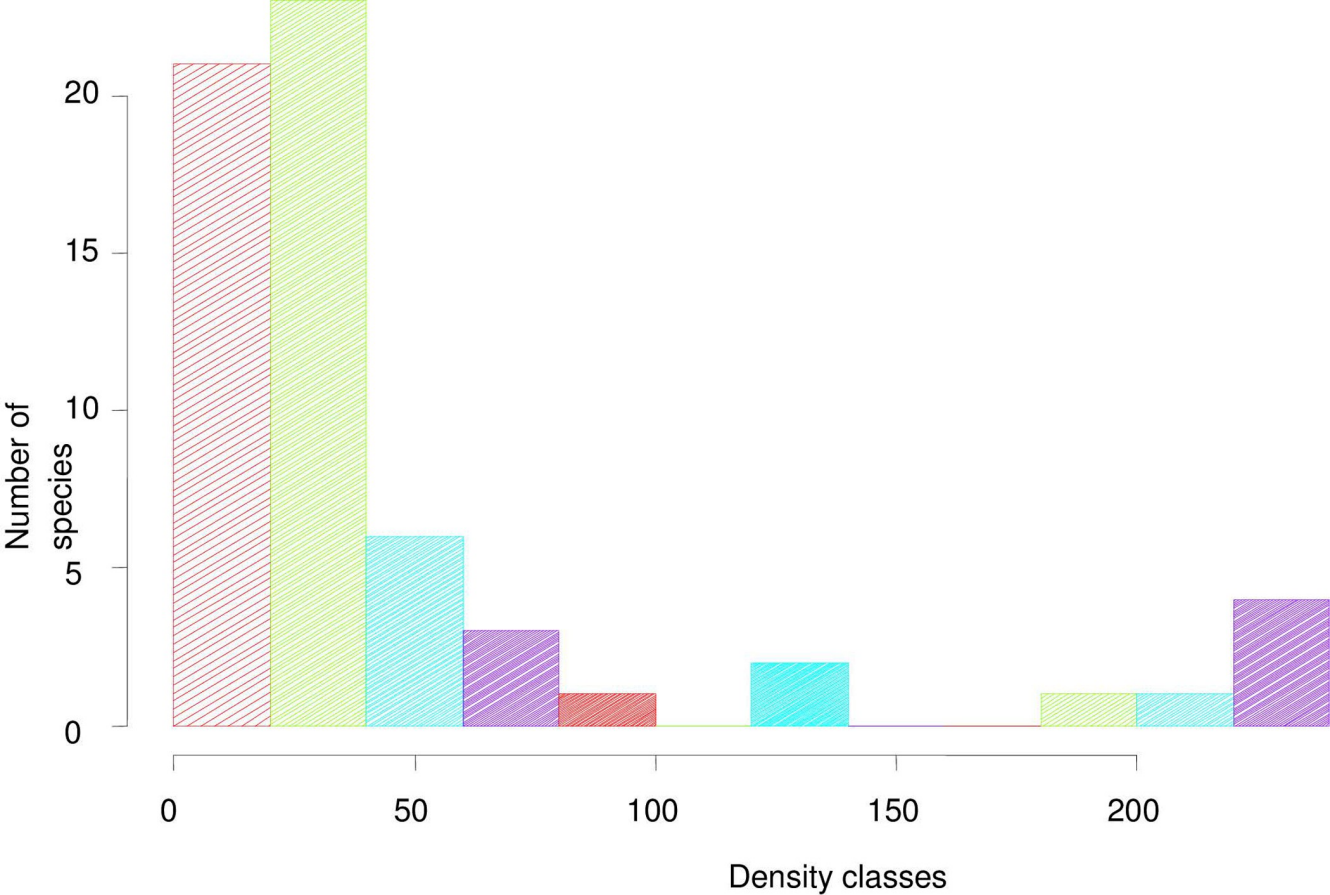
Species distribution across density classes.

As observed from the trend of the density classes (Fig 7), large numbers of species were distributed in the first density class followed by sharp decrease in the successive higher density classes. Such density class distribution revealed that most species were represented by small number of individuals while few species were represented by high number of individuals (>180 stems ha^-1^) in higher density classes (density class 7 and 8). Species with the highest density was *Pterolobium stellatum* (428.8 stems ha^-1^) followed b*y Dodonaea viscosa* subsp. *angustifolia (*404.5 stems ha^-1^), *Dichrostachys cinera* (335.1 stems ha^-1^) and *Euclea rasemosa (*329.1 stems ha^-1^) (S1 Table). The variation in density between species could be due to habitat preferences among the species, species characteristics for adaptation, degree of exploitation and conditions for regeneration [44, 54].

#### Diameter at Breast Height (DBH)

**Fig 8:**
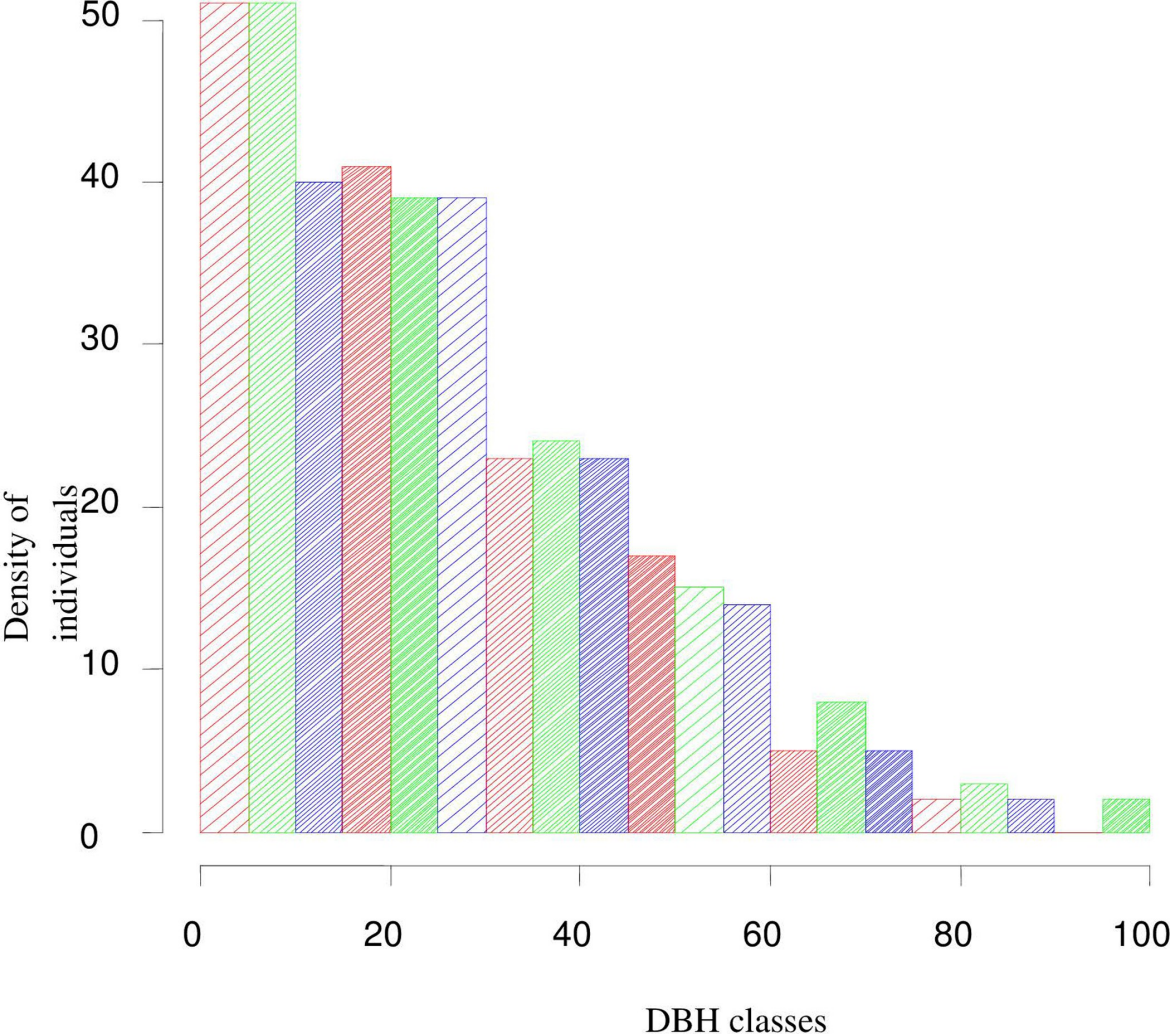
Distribution of individuals across DBH classes.

DBH distribution of individuals of species showed sharp decrease from DBH class 1 to class 2 followed by gradual declining tendency towards the higher DBH classes. Such pattern of individual distribution across the DBH classes is commonly known as inverted J-shape pattern (Fig 8) with the first class containing the largest proportion of stems ha^-1^ followed by continuous decreasing tendency towards the higher successive DBH classes (2^nd^, 3^rd^, 4^th^and 5^th^). The first DBH class contributed for about 69% of individuals ha^-1^. The remaining four DBH classes contributed 31% individuals ha^-1^ to the total individuals ha^-1^ of the forest. *Euclea rasemosa* (323 stems ha^-1^), *Pterolobium stellatum* (321 stems ha^-1^), *Premna schimperi* (222 stems ha^-1^), *Dodonaea viscosa* subsp. *angustifolia* (126 stems ha^-1^), *Grewia ferruginea* (124 stems ha^-1^*)* and *Calpurnia aurea* (118.8 stems ha^-1^) were the species that contributed much to the first DBH class.

The inverted J shape pattern, observed in the overall DBH classes of woody species was a representation of the normal vegetation structure and healthy regeneration of forest species. Such pattern is demonstrated by most remnant dry forests of Ethiopia [44, 46, 54, 68–70] which is an indicator of a forest species’ ability to regenerate and recruit normally [71].

#### Basal area (BA)

The overall basal area of Abraham Sacred forest was 35.18 m^2^ha^-1^. Five species, namely *Ficus thonningii* (5.3 m^2^ha^-1^), *Ficus sur* (4.42 m^2^ha^-1^), *Ficus vesta* (4.4 m^2^ha^-1^), *Olea europea* subsp. *cuspidata* (3.29 m^2^ha^-1^) and *Cordia africana* (3 m^2^ha^-1^) contributed a significant proportion of the basal area (S1 Table). The results demonstrated that a small number of species (5 species) contributed close to half of the total basal area. The total basal area of the forest was comparable to the typical basal area of tropical forests (35 m^2^ha^-1^) [72]. In addition, [18] in the sacred forests of South Gondar Zone (Amstya, 32.7 m^2^ha^-1^, Wonkesht, 34.2 m^2^ha^-1^, Gedamselase, 34.8 m^2^ha^-1^**),** [68] in Debre Libanos (33.46 m^2^ ha^-1^) and [45] in Gond (35.63 m^2^ha^-1^) disclosed a relatively similar basal area to the Abraham Monastery forest.

The higher total basal area in the Sacred forests is related to their sacredness whereby the forests are protected by religious sanctions and spiritual taught (community beliefs) that green matrix (surrounding) of religious places are the house of God that cannot be touched or disturbed. This makes the monastery/church forests the last refuge for hundreds of endangered, endemic and rare plant species. In addition, basal area of the forest indicates the status and age of the forest. Smaller basal area in some Monastery/church forests such as Enshete Kuskuam (18.5 m^2^ha^-^1) [18], Gatira Georges (7.84 m^2^ha^-1^) [73] and Arsema (9.16m^2^ha^-1^) [45] might be due to young age of the monastery where the forest is under early stage of succession.

*Importance value index (IVI)*. Tree species in Abraham Sacred forest were grouped into five classes based on their IVI value for conservation priority. The IVI classes and the number of species belonging to each class were described in Table 7. Although the representative number of specie in IVI class 5 is greater than that of classes 2, their IVI contribution is lower. The trend in the IVI percentage contribution showed an increase from class 2 to 4 and drastically dropped in IVI class 5 where the species in this class are at risk of local extinction. The result suggested that the tree species exhibit varying levels of dominance.

**Table 7:** IVI classes and numbers of species in each class of Abraham Monatery forest.

NO.	IVI class and values	Number of species	IVI total	Percentage
1	5(<1)	1	0.87	2.13
2	4(1–5)	34	65.45	72.3
3	3(5.1–10)	7	51.88	14.9
4	2(10.1–15)	5	59.58	10.64
5	1(>15)	-	-	-
	Total	47	177.75	100

As depicted in Table 8, the species with the highest IVI value was *Dodonaea viscosa* subsp. *angustifolia* (12.94) followed by *Dichrostachys cinera* (12.88), *Euclea rasemosa* (12.56), *Pterolobium stellatum* (10.98) and *Acokanthera schimperi* (10.22). The higher IVI value of these woody species was due to their higher relative density and frequency. Species with the least IVI due to their relative frequency, relative density and relative dominance were *Astropanax abyssinicus*, *Euphorbia abysinica* G.F. Gmel., *Syzygium guineense* (Willd.) DC., *Myrica salicifolia* and *Dombeya torrida*.

**Table 8:** Plant species arranged based on IVI value descending order including juveniles & adult density, Relative Frequency (RF), Relative Density (RD), and Relative Dominance (RDo) value of other species.

No	Species name	Density			RF	RD	RDo	IVI	Priority
		Seedling	Sapling	Adult					
1	*Dodonaea viscosa* subsp. *angustifolia*	122.8	179.2	102.5	8.8	0.39	3.75	12.94	1
2	*Dichrostachys cinera*	143.3	182.2	9.6	8.53	0.31	4.04	12.88	1
3	*Euclea racemosa*	101.2	152.5	75.43	8.5	0.23	3.83	12.56	1
4	*Pterolobium stellatum*	130.8	176.25	121.7	8.35	0.1	2.53	10.98	1
5	*Acokanthera schimperi*	120.8	94.86	80.4	6.45	0.17	3.6	10.22	1
6	*Cordia africana*.	12.5	13.3	10.41	6.12	0.45	3.39	9.96	2
7	*Calpurnia aurea*	120.5	40.83	30.4	4.7	0.91	3.16	8.77	1
8	*Grewia ferruginea*	135	53.75	32.9	5.16	0.13	3.32	8.61	1
9	*Prunus africana*	68.3	79.6	52.9	2.95	0.29	3.75	6.99	1
10	*Allophylus abyssinicus*	50.41	40.5	35.0	2.90	0.44	2.82	6.16	1
11	*Olea europaea* subsp. *cuspidata*	37.5	35.4	21.6	2.2	1.0	1.73	5.93	2
12	*Vachellia abyssinica*	18.75	12.1	10.4	0.94	0.42	1.37	5.46	2
13	*Croton macrostachyus*	15	19.6	21.25	1.3	0.48	2.2	3.98	2
14	*Bridelia micrantha* (Hochst.) Baill.	0	0	2.08	0.05	0.13	3.6	3.78	2
15	*Brucea antidysenterica*	6.67	12.1	11.9	0.7	0.58	2.24	3.52	2
16	*Ficus thonningii*	11.75	12.9	6.25	0.7	0.92	1.52	3.14	2
17	*Phytolacca dodecandra*	54.6	46.7	25.4	0.6	0.05	2.45	3.1	2
18	*Senegalia polyacantha*	12.5	18.75	12.25	1.0	0.34	1.59	2.93	2
19	*Damnacanthus indicus* var. *indicus*	175.4	47.1	40.83	0.84	0.39	1.59	2.82	1
20	*Combretum pisoniiflorum*	13.3	7.9	4.6	0.84	0.39	1.59	2.82	2
21	*Cobretum adenogonium*	2.08	37.5	25.0	0.72	0.25	1.8	2.77	2
22	*Ficus vasta*	9.6	6.25	3.75	0.45	1.3	1.01	2.76	2
23	*Ficus sur*	4.6	6.67	7.9	0.44	1.3	0.79	2.53	2
24	*Vachellia seyal* (Del.)P.J.H. Hurter	18.3	15.0	11.25	0.94	0.13	1.45	2.52	3
25	*Olea capensis*	4.1	7.9	20.7	0.76	0.14	1.56	2.46	2
26	*Ekebergia capensis*	22.1	18.33	12.5	1.23	0.47	0.72	2.42	3
27	*Erythrina abyssinica*	12.9	7.08	17.9	0.85	0.22	1.01	2.08	3
28	*Searsia glutinosa*	13.3	11.6	10	0.78	0.21	1.01	2	3
29	*Acacia hamiltoniana*	9.16	6.67	5.83	0.4	0.204	1.37	1.98	3
30	*Ximenia americana* L.	6.26	10.4	12.9	0.66	0.18	1.01	1.85	3
31	*Pyrex schimperiana*.(A. Rich.) Bridson	4.13	5	12.97	0.77	0.1	0.94	1.81	3
32	*Osyris lanceolata*	15.4	13.3	4.5	0.72	0.16	0.87	1.75	3
33	*Millettia ferruginea*.	9.6	5.8	3.33	0.44	0.17	1.08	1.69	3
34	*Erica arborea*	7.9	4.6	4.1	0.39	0.5	0.65	1.65	3
35	*Juniperus procera*	1.25	4.6	6.3	0.25	0.1	1.3	1.65	3
36	*Rosa abyssincia*	17.1	15	12.1	0.99	0.08	0.58	1.65	3
37	*Gardenia ternifolia*	10.8	6.25	2.5	0.45	0.4	0.79	1.64	4
38	*Adansonia digitata*	7.9	6.67	4.5	0.33	0.13	1.155	1.62	4
39	*Ziziphus spina-christi* Mill.	4.6	10.8	12.08	0.62	0.13	0.87	1.62	4
40	*Terminalia brownii*	7.9	15	1.67	0.55	0.15	0.79	1.49	4
41	*Stereospermum kunthianum* Cham.	6.25	5.8	4.6	0.37	0.25	0.72	1.34	4
42	*Premna schimperi*	4.1	5	12.9	0.55	0.1	0.65	1.3	3
43	*Dombeya torrida*	0.8	4.6	6.67	0.28	0.13	0.87	1.28	4
44	*Myrica salicifolia*	0	0	4.1	0.55	0.28	0.43	1.26	4
45	*Syzygium guineense*	0	0	2.9	0.07	0.17	0.94	1.18	4
46	*Euphorbia abyssinica*	2.5	2.1	1.66	0.14	0.23	0.72	1.09	4
47	*Astropanax abyssinicus*	7.9	3.3	2	0.3	0.17	0.4	0.87	4
48	*Acalypha psilostachya* var. *glandulosa Hochst*. *ex A*. *Rich*	4.5	5.83	3.75					
49	*Asparagus africanus* Lam.	6.67	13.5	7.9
50	*Calotropis gigantea var*. *procera* (Aiton) Dryand	8.75	29.1	1.67
51	*Capparis tomentosa*	14.1	10	2.9
52	*Cissus populnea*	12.15	17.08	13.3
53	*Clematis simensis* Fresen.	0	0	1.25
54	*Rotheca myricoides*	18.5	2.91	2.5
55	*Clutia abyssinica* Jaub. & Spach.	1.67	1.25	2.95
56	*Euphorbia tirucalli* L.	12.08	17.1	15
57	*Helinus mystacinus*	4.6	4.6	2.5
58	*Hetromorpha arborescens*	5.4	5	3.35
59	*Jasminum abyssinicum*	0	0	1.25
60	*Jasminum grandiflorum*	10	6.7	6.7
61	*Justicia schimperiana*	25	18.75	24.3
62	*Gymnosporia arbutifolia*	27.1	18.75	24.2
63	*Ocimum urticifolium* Benth.	6.25	3.33	2.5
64	*Opuntia ficus-barbarica*	4.6	6.3	7.9
65	*Ormocarpum pubescens*	13.3	7.1	9.6
66	*Rydinia integrifolia*	5.8	10	8.75
67	*Pavetta abyssinica*	15	10.4	7.1
68	*Rumex nervosus*	14.1	11.25	9.58
69	*Solanum giganteum Jacq*.	10	5	0
70	*Gymnanthemum amygdalinum* (Del.) Sch-Bip ex Walp.	15.3	8.75	5

Importance value index (IVI) reflects the degree of dominance and ecological importance of species relative to other co-occurring species in the community [40]. It gives a more realistic figure of dominance from the structural point of view and a species with the greatest importance value are the most dominant of the particular vegetation [67]. It might also be the most successful species in regeneration, pathogen resistance, growing in the shade, and in competition with other species, least preferred by browsers, high ability of attracting pollinators and predators that facilitate seed dispersal within the existing environmental conditions. Such species may not need immediate conservation measures but they need regular monitoring whereas species with lower IVI need conservation priority [45].

#### Regeneration status of Abraham Sacred Forest

The densities of seedlings (1801.1 individuals/ha) were greater than saplings (1678.1 individuals/ha) and trees (1100.9 individuals/ha), i.e. the density ratio of seedlings to saplings was 1.07: 1; seedling to mature 1.6:1 and sapling to mature 1.5:1. The result demonstrated that due to the large contribution of seedlings from certain species, the Abraham Sacred Forest appears to be in a good state of regeneration. *Damnacanthus indicus* var. *indicus*, *Grewia ferruginea*, *Acokanthera schimperi* and *Calpurnia aurea* accounted for the higher density of seedlings followed by saplings and trees (Table 8) that contributed much for the overall good regeneration status of the forest. Good regeneration status of the forest was also reported by [46] in Yemrehane Kirstos, [45] in Gond, [69] in Menfeskidus and [70] in Bradi Monastery forest.

However, separate analysis of regeneration status of species showed three regeneration patterns: 48.6% good, 20% fair and 7.1% none regenerating whereas previous studies [69, 70] reported the occurrence of four regeneration patterns (good, fair, poor and none regenerating). Several species (7.1%) including *Myrica salicifolia* and *Syzygium guineense* were none regenerating as they lacked seedlings and saplings (Table 8). Absence of regeneration might be attributed to anthropogenic pressure (selective cutting before seed production), lack of suitable seed bed for seed germination or problems associated with seed set (predation or abortion) and livestock grazing and trampling [70]. Therefore, the composition, distribution and density of seedlings and saplings within the forest are important indicators of its regeneration status. Thus, monitoring the presence and abundance of seedlings and saplings can provide valuable insights into the overall health and future sustainability of the forest ecosystem. Authors including [74] reported that the regeneration condition or recruitment condition of woody species is one of the major factors that are useful to assess their conservation status.

## Conclusion and recommendations

The presence of a diverse array of woody species in the forest suggests that it serves as an *in-situ* conservation site that has a potential role in protecting the native flora, contributing to the conservation efforts of the country’s indigenous plant species. Cluster analysis of floristic data generated three distinct communities that require tailored conservation strategies taking into account the specific ecological requirements and dynamics associated with different altitudes and slopes. A relatively, higher species diversity within the forest generally demonstrates a more stable and healthier condition of the ecosystem where multiple ecological niches are being occupied making the ecosystem more resilient to disturbances. The J-shaped pattern observed in frequency classes of species suggests the presence of vegetation heterogeneity that emphasizes the diverse ecological niches and species interactions within the forest ecosystem. In addition, the J-shaped pattern in DBH distribution of individuals indicates successful regeneration and recruitment of species contributing to the overall sustainability and future dynamics of the forest. The smaller basal area of larger number of species suggests that the forest is primarily composed of young individuals of woody species. IVI and regeneration data analyses revealed the presence of certain species with smaller IVI values and limited regeneration that highlights the vulnerability of species. These results, thus, underscore the importance of conservation efforts to protect and potentially restore the vulnerable species and maintain overall biodiversity within the ecosystem.

## Supporting information

S1 Table Species list collected from the study area and their occurrence, density and relative basal area. (DOCX)

## References

[1] Canton H. Food and agriculture organization of the United Nations—FAO. In The Europa directory of international organizations. Routledge, England; 2021. pp. 297–305.

[2] Millennium Ecosystem Assessment (2005). Ecosystems and human well-being. Island Press, Washington DC; 2005.

[3] Bank World. Poverty and the environment: Understanding linkages at the household level. The World Bank; 2007.

[4] Kumar P, Yashiro M. The marginal poor and their dependence on ecosystem services: evidence from South Asia and Sub-Saharan Africa. 2014. Pp.169–180.

[5] Brack D. Forests and climate change. In Proceedings of Background Study Prepared for the Fourteenth Session of the United Nations Forum on Forests. United Nations Forum on Forests. New York, USA, 2019.

[6] Salami A, Kamara AB, Brixiova Z. Smallholder agriculture in East Africa: Trends, constraints and opportunities. African Development Bank. Tunis, Tunisia. 2010.

[7] Kidane EE, Kiros S, Berhe A, Girma Z. Human-wildlife conflict and community perceptions towards wildlife conservation in and around a biodiverse National Park, northern Ethiopia. Global Ecology and Conservation. 2024; 54: 1–13.

[8] Tofu DA, Wolka K, Woldeamanuel T. The impact of alternative energy technology investment on environment and food security in northern Ethiopia. Scientific Reports. 2022; 12(1): 1–12. 35729275 10.1038/s41598-022-14521-2 PMC9213420

[9] Friis I, Demissew S, Breugel PV. Atlas of the potential vegetation of Ethiopia. The Royal Danish Academy of Sciences and Letters, Copenhagen, Denmark; 2010.

[10] Kassa D, Bekele A. Species composition, abundance, distribution and habitat association of rodents of Wondo Genet, Ethiopia. SINET: Ethiopian Journal of Science. 2008; 31(2): 141–146.

[11] Didita M, Nemomissa S, Gole TW. Floristic and structural analysis of the woodland vegetation around Dello Menna, Southeast Ethiopia. Journal of Forestry research. 2010; 21(4): 395–408.

[12] Kelbessa E, Demissew S. Diversity of vascular plant taxa of the flora of Ethiopia and Eritrea. Ethiopian Journal of Biological Sciences. 2014; 13(Supp.): 37–45.

[13] Tolessa T, Senbeta F, Kidane M. The impact of land use/land cover change on ecosystem services in the central highlands of Ethiopia. Ecosystem Services. 2017; 23: 47–54.

[14] Bekele T. Plant population dynamics of Dodonaea angustifolia and Olea europaea susp. cuspidata in dry Afromontane forests of Ethiopia. Acta Universitatis Upsaliensis, Uppsala; 2000. pp.1–47

[15] ViVero J L, Kelbessa E, Demissew S. The Red List of Endemic Trees & Shrubs of Ethiopia and Eritrea. Fauna & Flora International, Cambridge, UK; 2005. pp.1–28

[16] Stévart T, Dauby G, Lowry PP, Blach-Overgaard A, Droissart V, Harris DJ. et al. A third of the tropical African flora is potentially threatened with extinction. Science Advances. 2019; 5(11): 1–13. doi: 10.1126/sciadv.aax9444 31799397 PMC6867875

[17] Enquist BJ, Feng X, Boyle B, Maitner B, Newman EA, Jørgensen PM. et al. The commonness of rarity: Global and future distribution of rarity across land plants. Science advances. 2019; 5(11): 1–13 doi: 10.1126/sciadv.aaz0414 31807712 PMC6881168

[18] Wassie A, Sterck FJ, Bongers F. Species and structural diversity of church forests in a fragmented Ethiopian Highland landscape. Journal of Vegetation Science. 2010; 21(5): 938–948.

[19] Wassie A. Ethiopian church forests opportunities and challenges for restoration. Wageningen University and Research, the Netherlands; 2007.

[20] Tomar JMS, Ahmed A, Bhat JA, Kaushal R, Shukla G, Kumar R. Potential and opportunities of agroforestry practices in combating land degradation. Agroforestry-Small Landholder’s Tool for Climate Change Resiliency and Mitigation. 2021. pp.1–20

[21] Cheru GU, Hailu BK. Contribution of agroforestry practice in reducing deforestation and improving livelihood of household in Ethiopia. Journal of the Selva Andina Biosphere. 2023; 11(2): 172–185.

[22] Epple C, Thorley J. Options for REDD+ action: what are their effects on forests and people. An introduction for stakeholders in Central Sulawesi. UNEP-WCMC, Cambridge, UK; 2012.

[23] Nevin E. (2008). Education and sustainable development. Policy & Practice A Development Education Review. 2008; 6: 49–62.

[24] Meilani MM, Thwaites R, Race D, Andayani W, Faida LRW, Maryudi A. Finding alternatives of livelihood sources for forest dependent communities in protected areas: a case study of Sebangau National Park, Central Kalimantan Province, Indonesia. Earth and Environmental Science. 2019; 285 (1): 1–14.

[25] Tadese S, Soromessa T, Gebeyehu G. Effects of Environmental and Disturbance Factors on Plant Community Distribution in Tropical Moist Afromontane Forests, South-West Ethiopia. International Journal of Forestry Research. 2023: 1–1736741240

[26] McEwan RW, Dyer JM, Pederson N. Multiple interacting ecosystem drivers: toward an encompassing hypothesis of oak forest dynamics across eastern North America. Ecography. 2011; 34(2): 244–256.

[27] Melese GT, Tsegay BA, Kassa G.M. Effects of environmental variables on the patterns of plant community distribution in the afro-alpine vegetation of Simien Mountains National Park, Ethiopia. Journal of Biotechnology International. 2017; 10(1): 8–21.

[28] Krömer T, Acebey A, Kluge J, Kessler M. Effects of altitude and climate in determining elevational plant species richness patterns: a case study from Los Tuxtlas, Mexico. Flora-Morphology, Distribution, Functional Ecology of Plants. 2013; 208(3): 197–210.

[29] Zhu H, Yong C, Zhou S, Wang H, Yan L. (2015). Vegetation, floristic composition and species diversity in a tropical mountain nature reserve in southern Yunnan, SW China, with implications for conservation. Tropical Conservation Science. 2015; 8(2): 528–546.

[30] Bochet E, García‐Fayos P. Factors controlling vegetation establishment and water erosion on Motorway slopes in Valencia, Spain. Restoration ecology. 2004; 12(2): 166–174.

[31] Zeng XH, Zhang WJ, Song YG, Shen HT. (2014). Slope aspect and slope position have effects on plant diversity and spatial distribution in the hilly region of Mount Taihang, North China. Journal of Food Agriculture and Environment. 2014; 12: 391–397.

[32] Bekele T. Phytosociology and ecology of a humid Afromontane forest on the central plateau of Ethiopia. Journal of Vegetation Science. 1994; 5(1): 87–98.

[33] Van der Maarel E. Transformation of cover-abundance values in phytosociology and its effects on community similarity. Vegetatio. 1979; 39: 97–114.

[34] Woldu Zerihun, Feoli E. Lisanework Nigatu. Partitioning an elevational gradient of vegetation from southeastern Ethiopia. Vegetatio. 1989; 81: 189–198.

[35] Hickman KR, Hartnett DC, Cochran R, Owensby CE. Grazing management effects on plant species diversity in tall grass prairie. Journal of Range Managegment 2004; 57: 58–65.

[36] Heltshe JF, Forrester NE. (1983). Estimating species richness using the Jackknife procedure. Biometrics. 1983; 39(1): 1–11. 6871338

[37] Magurran A.E. (2004). Measuring Biological Diversity. Blackwell Publishing, Oxford, UK; 2004.

[38] Jongman RHG, Ter Braak CJ, Van Tongeren OF. (Eds.). Data analysis in community and landscape ecology. Cambridge University Press. 1995.

[39] Woldu Z. Environmental and Ecological Data analyses. Basics, concepts and methods. Lamberet Acadamic Puplishing, Addis Ababa, Ethiopia; 2012.

[40] Kent M. Vegetation description and data analysis: a practical approach. John Wiley & Sons, Chichester; 2011.

[41] McIntosh RP. Raunkiaer’s" law of frequency". Ecology. 1962; 43(3): 533–535.

[42] Dhaulkhand M, Dobhal A, Bhatt S, Kumar M. Community structure and regeneration potential of natural forest site in Gangotri, India. Journal of Basic and Applied sciences. 2008; 4(1): 49–52.

[43] Tiwari GPK, Tadele K, Aramde F, Tiwari SC. Community structure and regeneration potential of Shorearobusta forest in subtropical submontane zone of Garhwal Himalaya, India. Nature and Science. 2010; 8(1): 70–74.

[44] Zegeye H, Teketay D, Kelbessa E. Diversity and regeneration status of woody species in Tara Gedam and Abebaye forests, northwestern Ethiopia. Journal of Forestry Research. 2011; 22: 315–328.

[45] Masresha G, Lakew M, Chekole G. Comparative Study on Floristic Composition, Structure and Regeneration Status of Woody Plants: the Case of Gond Teklehaymanot and Arsema Monastery Forests, Amhara Region, Ethiopia. Ethiopian Journal of Natural Computational Sciences. 2021; 1(1): 13–36.

[46] Ayanaw A, Dalle G. Woody species diversity, structure, and regeneration status of Yemrehane Kirstos church forest of Lasta Woreda, North Wollo Zone, Amhara Region, Ethiopia. International Journal of Forestry Research. 2018: 1–9.

[47] Getnet A. Woody species composition, diversity and vegetation structure of dry Afromontane forest, Ethiopia. Journal of Agriculture and Ecology Research International. 2018; 16(3): 1–20.

[48] Wolde A. Review on Selected Church Forests of Ethiopia: Implication for Plant Species Conservation and Climate Change Mitigation. International Journal of Forestry Research, 202: 1–14.

[49] Muhando J. Sacred sites and environmental conservation: a case study of Kenya. Indilinga African Journal of Indigenous Knowledge Systems. 2005; 4(1): 228–242.

[50] Zeng L, Cao M, Lin L, Peters CM. (2022). Tree Diversity and Regeneration in Sacred Groves and Nature Reserves in Xishuangbanna, Southwest China. Journal of Ethnobiology. 2022; 42(4), 432–460.

[51] Ricklefs RE. Environmental heterogeneity and plant species diversity: a hypothesis. American Naturalist. 1977; 111(978): 376–381.

[52] Connell JH. Diversity in tropical rain forests and coral reefs. Science. 1978; 199(4335): 1302–1310. doi: 10.1126/science.199.4335.1302 17840770

[53] Baez Schon M, Woods CL, Cardelús L. (2022). Sacred Church Forests in Northern Ethiopia: Biodiversity and Cultural Islands. In: Montagnini F. (eds) Biodiversity Islands: Strategies for Conservation in Human-Dominated Environments. Topics in Biodiversity and Conservation. 2022. Pp.531–548.

[54] Abay K, Gebretsion K. Floristic composition, vegetation structure and regeneration status of Waldiba natural forest, northern Ethiopia. World Journal of Pharmaceutical and Life Sciences. 2020; 6 (1): 74–82

[55] Hedberg I, Kelbessa E, Edwards S, Demissew S, Persson E (Eds.). Flora of Ethiopia and Eritrea, Gentianaceae to Cyclocheilaceae. The National Herbarium, Addis Ababa University, Addis Ababa; 2006.

[56] Getaneh ZA, Demissew S, Woldu Z, Aynekulu E. (2023). Determinants of plant community along environmental gradients in Geramo forest, the western escarpment of the rift valley of Ethiopia. Plos one. 2023; 18(11): 1–15. 10.1371/journal.pone.0294324 PMC10681247 38011089

[57] Yigeremu A, Woldearegay M. Woody Species Diversity, Composition, and Regeneration Status of Abbo Sacred Forest, Southern Ethiopia. The Scientific World Journal. 2022: 1–9. doi: 10.1155/2022/9112578 36393830 PMC9652088

[58] Mekonnen AB, Wassie WA, Ayalew H, Gebreegziabher BG. Species Composition, Structure, and Regeneration Status of Woody Plants and Anthropogenic Disturbances in Zijje Maryam Church Forest, Ethiopia. Scientifica. 2022:1–14. doi: 10.1155/2022/8607003 36504490 PMC9733990

[59] Masresha G, Getnet A, Chekole G. Woody species composition, structure, and regeneration status of Gosh-Beret dry evergreen forest patch, South Gondar Zone, Northeast Ethiopia. International Journal of Forestry Research. 2023: 1–16.36741240

[60] Masresha G, Melkamu Y. The status of dry evergreen afromontane forest patches in Amhara National Regional State, Ethiopia. International Journal of Forestry Research. 2022(1): 1–11.

[61] O’Brien EM, Field R, Whittaker RJ. Climatic gradients in woody plant (tree and shrub) diversity: water‐energy dynamics, residual variation, and topography. Oikos. 2000; 89(3): 588–600.

[62] Pausas JG, Austin MP. Patterns of plant species richness in relation to different environments: an appraisal. Journal of Vegetation Science. 2001; 12(2): 153–166.

[63] Hailemariam MB, Temam TD. Pattern of plant community distribution along the elevational gradient and anthropogenic disturbance in Gole forest, Ethiopia. International Journal of Ecology. 2010; 2020: 1–9.

[64] Qin Y, Adamowski JF, Deo RC, Hu Z, Cao J, Zhu M. et al. Controlling factors of plant community composition with respect to the slope aspect gradient in the Qilian Mountains. Ecosphere. 2019; 10(9): 1–13.

[65] Birhanu L, Bekele T, Tesfaw B, Demissew S. Relationships between topographic factors, soil and plant communities in a dry Afromontane forest patches of Northwestern Ethiopia. PloS one. 2021; 16(3): 1–18. 10.1371/journal.pone.0247966 PMC7954303 33711027

[66] Tewolde BG. Vegetation and Environment of the Mountains in Ethiopia. Implications for Utilization and Observation. Mountain Research and development. 1987; 8: 211–216.

[67] Shibru S, Balcha G. Composition, Structure and regeneration status of woody species in Dindin Natural Forest, Southeast Ethiopia: An implication for conservation. Ethiopian Journal of Biological Sciences. 2004; 3(1): 15–35.

[68] Shiferaw W, Demissew S, Bekele T. Vegetation structure of Debra Libanos Monastery forest patch of north Oromia Region, Central Ethiopia. International License. 2019; 20: 135–150.

[69] Negesse G, Woldearegay M. Floristic diversity, structure and regeneration status of Menfeskidus Monastery forest in Berehet District, North Shoa, central Ethiopia. Trees, Forests and People. 2022; 7: 1–10.

[70] Yemata G, Haregewoien G. Floristic composition, structure and regeneration status of woody plant species in Northwest Ethiopia. Trees, Forests and People. 2022; 9: 1–10.

[71] Senbeta F, Schmitt C, Woldemariam T, Boehmer HJ, Denich M. Plant diversity, vegetation structure and relationship between plant communities and environmental variables in the Afromontane Forests of Ethiopia. SINET: Ethiopian Journal of Science. 2014; 37(2): 113–130.

[72] Midgleya JJ, Niklas KJ. Does disturbance prevent total basal area and biomass in indigenous forests from being at equilibrium with the local environment? Journal of Tropical Ecology. 2004; 20(5): 595–597.

[73] Ayalew A, Tesfaye A, Mulatu Y. Relation between regeneration status of woody species and soil properties of Gatira george’s Church and gemeshat remnant forests, north eastern Ethiopia. Black Sea Journal of Agriculture. 2020; 3(2): 110–119.

[74] Yeshitela K, Bekele T. The woody species composition and structure of Masha Anderacha forest, Southwestern Ethiopia. Ethiopian Journal of Biological Sciences. 2003; 2(1): 31–48.

