# Supplementary material for: Plant community and structural pattern analyses of Abraham Sacred Forest in Amhara Regional State, northwest Ethiopia
Source: PLoS One. 2025 Jan 14;20(1):e0317245. doi: 10.1371/journal.pone.0317245 (PMC11731766; doi:10.1371/journal.pone.0317245)
Supplement: S1 Table — (DOCX) [file pone.0317245.s001.docx]

**SI Table 1. Species list collected from the study forest and their occurrence, density and relative basal area**

| **Species** | **Family** | **H** | **F** | **D** | **RBA** |
| --- | --- | --- | --- | --- | --- |
| *Acacia hamiltoniana* Maid. | Fabaceae | T | 31.7 | 21.7 | 1.37 |
| *Acalypha psilostachya* Var. *glandulosa* Hutch. | Euphorbiaceae | S | 23.3 | 14.1 | 0.1 |
| *Acokanthera schimperi* (A.DC.) Schweinf. | Apocynaceae | T | 83.3 | 14.1 | 3.6 |
| *Adansonia digitata* L. | Bombacaceae | T | 26.7 | 27.5 | 1.15 |
| *Allophylus abyssinicus*  (Hochst.) Radlk. | Sapindaceae | T | 65 | 125.9 | 2.82 |
| *Asparagus africanus* Lam. | Asparagaceae | S | 41.6 | 28 | 0.3 |
| *Astropanax abyssinicus* (Hochst. ex A.Rich) Seem* | Araliaceae | T | 9.15 | 13.2 | 0.4 |
| *Bridelia micrantha* (Hochst.) Baill. | Euphorbiaceae | T | 8.3 | 2.1 | 3.6 |
| *Brucea antidysenterica J*.F. Mill. | Simarobaceae | T | 51.7 | 30.7 | 2.24 |
| *Calotropis gigantea var. procera* (Ait.) P.T. Li. | Asclepiadaceae | S | 35 | 39.5 | 0.23 |
| *Calpurnia aurea (Ait.)* Benth. | Fabaceae | S | 73 | 191.7 | 3.16 |
| *Capparis tomentosa* Lam. | Capparidaceae | S | 3.6 | 27 | 0.6 |
| *Cissus populnea* Guill. & Perr. | Vitaceae | L | 38.4 | 42.5 | 0.2 |
| *Clematis simensis* Fresen. | Ranunculaceae | L | 5 | 1.2 | 0.2 |
| *Clutia abyssinica* Jaub. & Spach. | Euphorbiaceae | S | 13.4 | 5.9 | 0.1 |
| *Combretum adenogonium* Steud. ex A. Rich | Combretaceae | T | 41.6 | 64.6 | 1.8 |
| Combretum pisoniiflorum (Klotzsch) Engl. | Combretaceae | T | 45 | 28.8 | 1.59 |
| *Cordia africana* Lam. | Bignoniaceae | T | 36.7 | 36.2 | 3.39 |
| *Croton macrostachyus* Hochest. ex Del. | Euphorbiaceae | T | 50 | 2.1 | 2.2 |
| *Damnacanthus inicus* var. *indicus* C.F. Gaertn | Apocynaceae | S | 78.34 | 263.3 | 1.59 |
| *Dichrostachys cinerea* (L.)Wight & Arn. | Fabaceae | T | 93.3 | 335.1 | 4.04 |
| Dodonaea viscosa subsp. *angustifolia* (L.f.)J.G. West | Sapindaceae | S | 86.7 | 404.5 | 3.75 |
| *Dombeya torrida* (J. F.Gmel.) P. Bamps | Sterculiaceae | S | 20 | 12.1 | 0.87 |
| *Ekebergia capensis* Sparrm. | Meliaceae | T | 16.7 | 52.9 | 0.72 |
| *Erica arborea* L. | Ericaceae | T | 15 | 16.6 | 0.65 |
| *Erythrina abyssinica* Lam. | Fabaceae | T | 23.3 | 37.9 | 1.01 |
| *Euclea racemosa L.* | Ebenaceae | S | 88.3 | 329.1 | 3.83 |
| *Euphorbia chamaesyce L* | Euphorbiaceae | T | 16.7 | 6.1 | 0.22 |
| *Euphorbia tirucalli L.* | Euphorbiaceae | S | 31.7 | 44.1 | 0.11 |
| *Ficus sur Forssk.* | Moraceae | T | 11.6 | 19.2 | 0.79 |
| *Ficus thonningii Blume.* | Moraceae | T | 35 | 30.9 | 1.52 |
| *Ficus vasta Forssk.* | Moraceae | T | 23.4 | 19.6 | 1.01 |
| *Gardenia ternifolia* Schumach. & Thonn. | Rubiaceae | T | 18.3 | 19.6 | 0.79 |
| *Grewia ferruginea* Hochst. ex A. Rich. | Malvaceae | S | 76.6 | 221.6 | 3.32 |
| Gymnanthemum *amygdalinum* (Delile) Sch.Bip | Asteraceae | S | 18.3 | 29.1 | 0.2 |
| *Gymnosporia arbutifolia* (Hochest, ex A. Rich.) Leos. | Celastraceae | S | 25 | 70.1 | 0.82 |
| *Helinus mystacinus* (Ait.) E. Mey. ex Steud. | Rhamnaceae | L | 23.4 | 11.7 | 0.1 |
| *Heteromorpha arborescens* (Spreng.) Cham. & Schltdl. | Apiaceae | S | 5 | 13.75 | 0.1 |
| *Jasminum abyssinicum* Hochst. ex DC. | Oleaceae | L | 26.7 | 1.3 | 0.1 |
| *Jasminum grandiflorum* L. | Oleaceae | L | 13.3 | 23.4 | 0.1 |
| *Juniperus procera* Hochst. ex Endl. | Cupressaceae | T | 30 | 12.2 | 1.3 |
| *Justicia schimperiana* T. Anderson | Acanthaceae | S | 11.6 | 68.1 | 0.3 |
| *Millettia ferruginea (*Hochst.) Hochst. Baker | Fabaceae | T | 10 | 18.7 | 1.08 |
| *Myrica salicifolia Hochest. ex.* A. Rich.* | Myrtaceae | T | 15 | 4.1 | 0.43 |
| *Ocimum basilicum L.* | Lamiaceae | S | 26.7 | 12.1 | 0.3 |
| *Olea capensis* L. | Oleaceae | T | 40 | 32.7 | 1.56 |
| *Olea europaea* subsp. *cuspidata* (Wall. & G. Don.) Cif*.* | Oleaceae | T | 20 | 94.5 | 1.73 |
| *Opuntia ficus-barbarica* A. Berger. | Cactaceae | S | 15 | 18.8 | 0.5 |
| *Ormocarpum pubescens* (Hochst.) Cufod. ex G.B. Gillett | Fabaceae | S | 20 | 30 | 0.31 |
| *Osyris lanceolata* Hochst. & Steud | Santalaceae | S | 11.7 | 33.2 | 0.87 |
| *Pavetta abyssinica* Fresen. | Rubiaceae | S | 18.3 | 32.5 | 0.31 |
| *Phytolacca dodecandra* L’Hér. | Phytolaccaceae | L | 18.3 | 126.7 | 2.45 |
| *Premna schimperi* Engl. | Verbenaceae | S | 56.7 | 22 | 0.65 |
| *Prunus africana* (Hook. f.) Kalkm. | Rosaceae | T | 15 | 200.1 | 3.75 |
| *Psydrax schimperiana* subsp. *accidentalis Br.** | Rubiaceae | T | 9.15 | 1.1 | 0.1 |
| *Pterolobium stellatum* (Forssk.) Brenan*.* | Fabaceae | L | 86.7 | 428.8 | 2.53 |
| *Rosa abyssinica R.Br.* ex. Lindl. | Rosaceae | S | 58.3 | 44.2 | 0.58 |
| *Rotheca myricoides* (Hochst.) Steane & Mabb. | Lamiaceae | S | 23.3 | 23.9 | 0.3 |
| *Rumex nervosus* Vahl. | Polygonaceae | S | 23.3 | 34.9 | 0.2 |
| *Rydingia integrifolia* (Benth.) Sch. &V.A. Albert | Lamiaceae | S | 22.5 | 24.6 | 0.22 |
| *Searsia glutinosa (*Hochst. ex. A. Rich.) Moff. | Anacardiaceae | T | 21.7 | 34.9 | 1.01 |
| *Senegalia polyacantha (*Willd.) Seigler. & Ebinger | Fabaceae | T | 36.7 | 43.5 | 1.59 |
| *Solanum giganteum* Jacq | Solanaceae | S | 16.7 | 15 | 0.2 |
| *Stereospermum kunthianum* Cham. | Bignoniacea | T | 9.2 | 16.7 | 0.72 |
| *Syzygium guineense* (Willd.) DC. | Myrtaceae | T | 11.7 | 2.9 | 0.94 |
| *Terminalia brownii*  Fressn*.* | Combretaceae | T | 21.6 | 24.6 | 0.79 |
| *Vachellia*  *abyssinica (*Hochst. ex Benth.) Kyal. & Boatwr. | Fabaceae | T | 31.7 | 41.3 | 1.37 |
| *Vachellia*  *seyal* Del. P.J.H. Hurter | Fabaceae | T | 33.4 | 44.6 | 1.45 |
| *Ximenia americana* L. | Oleaceae | S | 21.7 | 29.6 | 1.01 |
| *Ziziphus spina-christi* (L.) Desf. | Rhamnaceae | T | 23.3 | 27.5 | 0.79 |

Key: H = habit, L = liana, S = shrub, T = tree, Rf, = relative density, D = density, RBA = relative basal area
